# Lipopolysaccharides from *Ralstonia solanacearum* induce a broad metabolomic response in *Solanum lycopersicum*

**DOI:** 10.3389/fmolb.2023.1232233

**Published:** 2023-08-10

**Authors:** Dylan R. Zeiss, Antonio Molinaro, Paul A. Steenkamp, Alba Silipo, Lizelle A. Piater, Flaviana Di Lorenzo, Ian A. Dubery

**Affiliations:** ^1^ Research Centre for Plant Metabolomics, Department of Biochemistry, University of Johannesburg, Auckland Park, South Africa; ^2^ Department of Chemical Sciences, University of Napoli Federico II, Complesso Universitario Monte Sant’Angelo, Naples, Italy; ^3^ Task Force on Microbiome Studies, University of Napoli Federico II, Complesso Universitario Monte Sant’Angelo, Naples, Italy

**Keywords:** hydroxycinnamic acid amides, lipopolysaccharides, oxylipins, phenylpropanoids, plant immunity, metabolomics, *Solanum lycopersicum*

## Abstract

*Ralstonia solanacearum,* one of the most destructive crop pathogens worldwide, causes bacterial wilt disease in a wide range of host plants. The major component of the outer membrane of Gram-negative bacteria, lipopolysaccharides (LPS), has been shown to function as elicitors of plant defense leading to the activation of signaling and defense pathways in several plant species. LPS from a *R. solanacearum* strain virulent on tomato (LPS_*R. sol*._), were purified, chemically characterized, and structurally elucidated. The lipid A moiety consisted of tetra- to hexa-acylated *bis*-phosphorylated disaccharide backbone, also decorated by aminoarabinose residues in minor species, while the O-polysaccharide chain consisted of either linear tetrasaccharide or branched pentasaccharide repeating units containing α-L-rhamnose, *N*-acetyl-β-D-glucosamine, and β-L-xylose. These properties might be associated with the evasion of host surveillance, aiding the establishment of the infection. Using untargeted metabolomics, the effect of LPS_*R. sol.*_ elicitation on the metabolome of *Solanum lycopersicum* leaves was investigated across three incubation time intervals with the application of UHPLC-MS for metabolic profiling. The results revealed the production of oxylipins, e.g., trihydroxy octadecenoic acid and trihydroxy octadecadienoic acid, as well as several hydroxycinnamic acid amide derivatives, e.g., coumaroyl tyramine and feruloyl tyramine, as phytochemicals that exhibit a positive correlation to LPS_*R. sol.*_ treatment. Although the chemical properties of these metabolite classes have been studied, the functional roles of these compounds have not been fully elucidated. Overall, the results suggest that the features of the LPS_*R. sol.*_ chemotype aid in limiting or attenuating the full deployment of small molecular host defenses and contribute to the understanding of the perturbation and reprogramming of host metabolism during biotic immune responses.

## 1 Introduction

The sessile nature of plants exposes them to a range of opportunistic pathogens within the surrounding environment. The evolution-driven immunity of plants has, however, bestowed the ability of perceiving selective pathogen-derived molecules, so called microbe-assisted molecular patterns (MAMPs) through membrane bound pattern recognition receptors (PRRs) that result in the activation of various intracellular signaling pathways leading to the activation of an innate immune response known as MAMP-triggered immunity (MTI). *Ralstonia solanacearum* is a Gram-negative soil-borne pathogen with a high destructive capacity, causing bacterial wilt disease and severe crop losses in 310 plant species belonging to 42 families (Wang et al., 2023). However, little is known about the perception of immune elicitors from *R. solanacearum* (Shi et al., 2023)*.* A consequence of this is that agricultural approaches, to maintain the bacterial wilt disease, have been limited. The main surface component of the outer membrane of Gram-negative bacteria is a permeable barrier composed of lipopolysaccharides (LPS). This family of amphipathic molecules protects cells from harmful substances within the surrounding area, allowing the growth and development of the pathogen in unfavorable environmental stress conditions (Alexander and Rietschel, 2001; Li et al., 2014; Kutschera and Ranf, 2018).

As a tripartite saccharolipid/lipoglycan, LPS is a heat-stable amphiphilic molecule that consists of the O-polysaccharide (OPS) chain, a core oligosaccharide (COS), and the lipid A component. The first domain, the hydrophobic membrane-anchored lipid A, typically consists of a glucosamine (GlcN) dimer, *N*- and *O*-substituted with hydroxy fatty acids of variable number chain length (Newman et al., 2001; Di Lorenzo et al., 2022). The composition of the lipid A molecule has been shown to remain largely conserved among related Gram-negative bacterial species. The second domain consists of the COS, and the third domain consists of the OPS. These saccharide portions are the most structurally diverse components of an LPS, with the OPS displaying the greatest structural variability as it is formed by a variable number of oligosaccharide repeats. Of note, the cross-linking of the negatively charged residues, often present in the COS and lipid A, through the action of divalent cations (Ca^2+^/Mg^2+^) facilitates the tight packaging of the LPS (Ranf, 2016). This phenomenon is essential for the rigidity and low permeability of the outer bacterial membrane. LPS containing all three of the components mentioned previously are commonly referred to as ‘smooth’ LPS (S-LPS). The OPS is not a crucial component required for cell viability, and mutants lacking this component are often referred to as ‘rough’ (which have a rough LPS, R-LPS) (Newman et al., 2001; Li et al., 2014; Di Lorenzo et al., 2022), due to the morphological appearance of the colonies compared to that of the wild-type counterparts. It has already been shown that defective bacterial mutants lacking the OPS are vital but lack pathogenicity, display increased sensitivity to antibiotic and antimicrobial substances, and decline rapidly upon introduction in plants (de Castro et al., 2010; Erbs et al., 2010; Rapicavoli et al., 2018). However, some other studies reported that the OPS is not the active moiety in triggering plant defense responses (Newman et al., 2001; Caroff and Karibian, 2003; Esposito et al., 2008; Di Lorenzo et al., 2022).

During the infection process, LPS molecules form a physical barrier, protecting the bacterium from the cytotoxic effects of plant-derived antimicrobial phytochemicals to promote pathogenesis. As a part of innate immunity, the pathogen can be sensed by the perception of several pathogen-derived molecular signatures, e.g., flagellin-peptides and peptidoglycan. LPS derived from the bacterial coat can similarly act as an MAMP and trigger a host immune response (Madala et al., 2011; Mareya et al., 2020). These functional contradictions raise the scientific question about the pathogen’s method of successfully infecting the host plant (Aslam et al., 2008) and strategies to evade plant immunity (Wang et al., 2022). To date, no dedicated pattern recognition receptor for bacterial LPS has been discovered in plants. An untargeted metabolomics approach was followed to obtain greater understanding surrounding the LPS-induced defense-related metabolic reprogramming that occurs in leaf tissues of *Solanum lycopersicum* (tomato). This is believed to be the first report on the metabolomic dynamics associated with the perception of LPS_*R. sol.*_ in *S. lycopersicum*.

## 2 Materials and methods

### 2.1 Plant cultivation conditions

The STAR9008 (8SC) tomato cultivar seeds were obtained from a tomato breeding program associated with resistance against *R. solanacearum* (Stark Ayres, Pty. Ltd., Bredell, South Africa, www.starkeayres.co.za). The seeds were germinated in a commercially available germination soil mixture (Culterra, Muldersdrift, South Africa). The tomato plants were grown under greenhouse conditions: a light/dark cycle of 12 h/12 h, with the light intensity set at 80 μmol/m^2^/s, and the temperature regulated between 22°C and 24°C. The plants were 30–35 cm in height at the time of the experiments.

### 2.2 *Ralstonia solanacearum*, cell culture, and extraction of LPS

The strain of *R. solanacearum* used in this study was BD 261 (Coutinho collection, University of Pretoria, GenBank Accession number KY 709230) from South African origin belonging to race 2 biovar 3. Prior to cultivation, the pathogen was plated out on both the triphenyl tetrazolium chloride (TTC) media and the selective South Africa-Elphinstone (SMSA-E) media at 28°C for 48 h (Pontes et al., 2017). The plating step was added to monitor colony purity and to prevent external contamination. This strain was used in previous studies regarding the metabo-phenotyping and metabolomics of the host response (disease tolerance) of the selected tomato cvs. (Zeiss et al., 2018; 2019) and the inducible changes of the metabolome to peptide elicitors derived from *R. solanacearum* flagellin (Zeiss et al., 2021a) and cold shock protein (Zeiss et al., 2022). LPS was purified from saturated cell cultures grown in the nutrient broth medium on an orbital shaker at 28°C, using the hot phenol protocol as previously described (Gerber et al., 2004) and incorporating DNase and protease digestion steps (Madala et al., 2011).

### 2.3 Gel electrophoresis of isolated LPS

Sodium dodecyl sulfate-polyacrylamide gel electrophoresis (SDS-PAGE) was performed to evaluate the type of LPS and size heterogeneity within the LPS fraction. Purified LPS_*R. sol.*_ was dissolved in distilled water (1 mg/mL stock). LPS from *Burkholderia cepacia*, (LPS_*B.cep.*_) (Gerber et al., 2004; Madala et al., 2011) was used as a positive control. Equal volumes of the sample and a 2X sample buffer, consisting of 125 mM Tris-Cl pH 6.8, 4% SDS, 20% glycerol, 10% β-mercaptoethanol, and 0.02% bromophenol blue were mixed. The combined sample was placed on a heating block set at 95°C for about 2 min and left to cool. The gel (100 mm × 80 mm × 0.75 mm) was run at a constant voltage (90 V) with varying current (≤40 mA) and subsequently stained using a silver staining protocol (Gerber et al., 2004). Briefly, the gel was fixed in a solution containing 40% ethanol (v/v) and 10% acetic acid (v/v) for 30 min. Following the fixing step, the gel was washed with distilled water for 15 min, which was repeated 2–3 times. The gel was immersed in a 0.5% periodic acid solution for 2 min with constant agitation. The gel was again washed with distilled water for 30 min, which was repeated 2–3 times. The gel was then immersed in a staining solution composed of 0.02% silver nitrate (AgNO_3_) and 1 mM formaldehyde for 30 min with constant agitation. Excess staining solution was then rinsed off with distilled water followed by the addition of the developing solution composed of 14% Na_2_CO_3_.10H_2_O, 20 mM Na_2_S_2_O_3_.5H_2_O, and 6 mM formaldehyde. Upon visualization of the bands on the gel, the reaction was halted with the addition of 7.5% acetic acid for 15 min, followed by washing the gel with distilled water for 30 min. The gel was then photographed and recorded.

### 2.4 Chemical (GC-MS) analysis of *Ralstonia solanacearum* LPS

For structural elucidation, additional steps of purification by ultracentrifugation (100,000 × *g*, 16 h, 4°C) and size-exclusion chromatography on a Sephacryl S-500 column were performed. The LPS monosaccharide content was established by the inspection of the acetylated *O*-methyl glycoside derivatives (AMG) obtained by treatment with HCl/MeOH (1.25 M, 85°C, 16 h) followed by acetylation with acetic anhydride in pyridine (85°C, 20 min). The absolute configuration was established through the evaluation of the acetylated *O*-octylglycoside (OGA) derivatives and comparison with authentic standards, as previously described (Leontein, 1978; Garcia-Vello et al., 2022). To define the sugar linkage pattern, an aliquot of dried sample was suspended in dimethylsulfoxide (DMSO) in the presence of a spatula tip of NaOH with alternating stirring and sonication for 2 h at room temperature, before treatment with iodomethane. Then, it was hydrolyzed with trifluoroacetic acid (4 M, 100°C, 4 h), carbonyl reduced with NaBD_4_ and acetylated with pyridine and acetic anhydride (Ciucanu and Kerek, 1984; Garcia-Vello et al., 2022). The so-obtained partially methylated alditol acetates (PMAAs), as well as AMG and OGA, were then analyzed by gas chromatography with mass spectrometry detection (GC–MS).

The total fatty acid content was established by treating with HCl (4 M, 100°C, and 4 h) followed by a treatment with NaOH (5 M, 100°C, and 30 min). The pH was adjusted to reach slight acidity (pH ⁓3). After extraction in chloroform, fatty acids were methylated with diazomethane and inspected via GC–MS. The absolute configuration of hydroxy fatty acids was determined as previously reported (Rietschel, 1976). Briefly, the hydroxy fatty acids were released after the treatment with 4 M NaOH (100°C and 4 h), converted into 3-methoxy acid L-phenylethylamides, and then analyzed by GC–MS. The comparison of the retention times (Rts) of authentic L-phenylethylamides of standard fatty acids with those derived from the examined LPS, allowed the assignment of the (*R*) configuration to all of the 3-hydroxy fatty acids and the (*S*) configuration to all of the 2-hydroxy fatty acids composing the lipid A of *R. solanacearum* LPS. All chemical analyses were executed through the employment of an Agilent Technologies Gas Chromatograph 7820A equipped with a mass selective detector 5977B and an HP-5 capillary column (Agilent, Milan, Italy, 30 m × 0.25 mm i.d., flow rate 1 mL/min, He as carrier gas). The temperature program used to analyze AMG and OGA was 140°C for 3 min and then 140 → 240°C at 3°C/min. The temperature program for PMAA was 90°C for 1 min, 90 →140°C at 25°C/min, 140 → 200°C at 5°C/min, 200 → 280°C at 10°C/min, and finally 280°C for 10 min. To analyze the fatty acid content, the following temperature program was used 150°C for 5 min, 150°C–280°C at 3°C/min, and 280°C for 5 min.

### 2.5 Isolation of lipid A and OPS from *Ralstonia solanacearum* LPS by MALDI-TOF MS

The purified LPS (30 mg) were treated with acetate buffer solution (pH 4.4, 100°C, 2 h). After centrifugation (4,000 × *g*, 30 min), the supernatant containing the OPS fraction was collected and lyophilized. The OPS was then purified by size exclusion chromatography on a Toyopearl TSK HW-40S (Tosoh Bioscience, 1.5 × 100 cm, eluent 50 mM NH_4_HCO_3_) column. The precipitate, containing lipid A, was collected and washed several times with freshly prepared Bligh/Dyer mixture (chloroform/methanol/water, 2:2:1.8, v/v/v) (Bligh and Dyer, 1959). The organic phases were pooled, dried, and analyzed by matrix-assisted laser desorption ionization time-of-flight mass spectrometry (MALDI-TOF MS).

### 2.6 NMR spectroscopy analysis of the O-polysaccharide chain

1D and 2D NMR spectra were recorded on a Bruker 600 AVANCE NEO instrument. The solvent was D_2_O. Spectra calibration was performed with internal acetone (*δ*_H_ 2.225 ppm, *δ*_C_ 31.45 ppm). The double-quantum-filtered phase sensitive correlation spectroscopy (DQF-COSY) experiment was carried out by using datasets of 4,096 × 256 points (Piantini et al., 1982; Rance et al., 1983). Total correlation spectroscopy (TOCSY) experiments were performed with spinlock times of 100 m, using datasets (t1 × t2) of 4,096 × 256 points. Nuclear Overhauser Enhancement Spectroscopy (NOESY) experiment was recorded by using datasets (t1 × t2) of 4,096 × 256 points and by applying mixing times between 100 and 400 m. In all homonuclear experiments, the data matrix was zero-filled in both dimensions to give a matrix of 4 K × 2 K points and was resolution-enhanced in both dimensions by a cosine-bell function before Fourier transformation (Speciale et al., 2022). The determination of coupling constants was obtained by 2D phase-sensitive DQF-COSY. Heteronuclear single quantum coherence (^1^H, ^13^C HSQC) and heteronuclear multiple bond correlation (^1^H, ^13^C HMBC) experiments were recorded in the ^1^H-detection mode by single-quantum coherence with proton decoupling in the ^13^C domain using datasets of 2048 × 256 points. ^1^H, ^13^C HSQC was performed using sensitivity improvement in the phase-sensitive mode using echo/antiecho gradient selection, with multiplicity editing during the selection step. The ^1^H, ^13^C HMBC experiment was optimized on long-range coupling constants with low-pass J filter to suppress one-bond connectivity, using gradient pulses for selection. A delay of 60 m was employed for the evolution of long-range correlations. It was used at a long-range coupling constant value of 6 Hz. The data matrix in both heteronuclear experiments was extended to 2048 × 1024 points using forward linear prediction extrapolation (Stern et al., 2002).

### 2.7 MALDI-TOF mass spectrometry analysis

MALDI-TOF MS spectra were recorded on an AB SCIEX TOF/TOF™ 5800 Applied Biosystems mass spectrometer equipped with an Nd:YAG laser (λ = 349 nm), with a 3 ns pulse width and a repetition rate of up to 1,000 Hz. The lipid A fraction was dissolved in CHCl_3_/CH_3_OH (1:1, v/v). The matrix solution was 2,4,6-trihydroxyacetophenone in CH_3_OH/0.1% trifluoroacetic acid/CH_3_CN (7:2:1, v/v) at a concentration of 75 mg/mL (Di Lorenzo, 2017; Mareya et al., 2020). 0.5 μL of the sample and 0.5 μL of the matrix solution were deposited on the MALDI plate and left to dry at room temperature. The lipid A and matrix solutions were spotted in triplicate on the MALDI plate. For MS experiments, each spectrum was a result of the accumulation of 2,000 laser shots, whereas 5,000–7,000 shots were summed for the MS/MS spectra.

### 2.8 Reactive oxygen production: histochemical staining and luminescence assay

The leaves of mature tomato plants were treated with the LPS_*R. sol.*_ elicitor and were stained with a 3,3′-diaminobenzidine (DAB), (Sigma-Aldrich, St. Louis, United States) solution to visually detect elicitor-linked hydrogen peroxide production. The protocol was performed with minor modifications to that of Bach-Pages and Preston (2018). Briefly, the left abaxial side of the leaves was pressure-infiltrated with 100 μg/mL LPS_*R. sol.*_ with a blunt-ended syringe, while the right abaxial half was treated with 8 mM MgSO_4_ as a negative control. During the pressure infiltration process, care was taken to limit wounding or mechanical damage to the leaf tissues. The DAB solution (1 mg/mL in water, pH 3.8) was made up prior to inoculation and was covered with foil. The inoculated leaves were excised from the plant, immersed in the DAB solution, and left to incubate with constant agitation at 23°C for 8 h. Following the incubation interval, the leaves were removed and immersed in boiling 70% ethanol for 10 min and then transferred into absolute ethanol at 37°C and left overnight. The visible presence of the brown polymerized precipitate in the host tissue indicated a reaction between DAB and H_2_O_2_.

For the luminescence assay, a cork borer was used to punch out 0.4 cm^2^ leaf disks from fully expanded tomato leaves above the fourth node. The disks were floated on 200 µL MilliQ water in a white 96-well microtiter plate (Nunc, Roskilde, Denmark) with the adaxial side up. The leaf disks were placed under light at 37°C and left to incubate for 24 h. After the incubation period, water was completely removed and replaced with a 100 µL of a master mix solution composed of 34 μg/mL luminol, 20 μg/mL horseradish peroxidase (Sigma-Aldrich, St. Louis, United States), and 100 μg/mL LPS_*R. sol.*_ in water. Special care was taken to limit the damage of the leaf disks during the floating disk and water removal steps. A negative control composed of the abovementioned master mix—excluding the LPS elicitor and supplemented with water—was added. Luminescence was measured every 2 min for 60 min using a Synergy HT BioTek microplate reader (Biotek Instruments, Vinooski, VT, United States). The luminescence data were exported for further analysis. To account for natural variability, three leaf disks per plant were taken. Overall, the procedure was repeated as three independent experiments.

### 2.9 Plant elicitation and experimental design for metabolomic analysis

The tomato plants (selected based on uniformity in size and appearance) were watered 5 h prior to the inoculation step, to open leaf stomata and ease the process of pressure infiltration. The layout of the treatment process was as follows: three tomato plants were reserved for each of the LPS_*R. sol.*_ treatments at the respective time intervals (16, 24, and 32 h), along with the addition of corresponding MgSO_4_ controls. Sampling consistency was maintained by selecting the leaves from the fourth node for LPS_*R. sol.*_ or control treatment.

The LPS stock (1 mg/mL) was dissolved in sterile 8 mM MgSO_4_ to a working concentration of 100 μg/mL. The plants were treated with 100 μg/mL LPS_*R. sol.*_ by pressure infiltration into the abaxial side of the leaves using a blunt-ended syringe. Again, the control group was inoculated with an 8 mM MgSO_4_ solution. The plants selected for each of the conditions were treated in separate locations to prevent cross-contamination. During the pressure infiltration step, the entire leaf surface was supplied with the appropriate elicitor/control solution, which minimized the level of biological variation and permitted the harvest of the entire leaf tissue. Care was taken to avoid/limit wounding or mechanical damage. After inoculation, the cultivar groups were left to incubate for 16, 24, and 32 h, respectively. Following the incubation times, the inoculated leaves for each group condition were harvested, and metabolic activity was quenched in liquid nitrogen and stored at −80°C.

The experimental design included three independent biological replicates. Sufficient plant material was thus obtained to create a total of three biological replicates for each of the control and elicitor-treatment groups across the three incubation intervals. Together with three analytical replicates of each, this generated *n* = 9 as required for metabolomic analysis based on multivariate statistics (Sumner et al., 2007; Tugizimana et al., 2013).

### 2.10 Metabolite extraction and sample preparation

For the extraction procedure, the leaf tissue stored at −80°C was submerged in liquid nitrogen and pulverized with a mortar and pestle. 2 g of leaf powder from each of the group conditions were extracted with cold 80% methanol in a 1:10 (w/v) ratio. The samples were sonicated with both a sonicator probe (Bandelin Sonopuls, Berlin, Germany), set at 100% power for 30 s, and a sonicator bath for 30 min at 15°C. The samples were centrifuged using a bench-top swinging-bucket centrifuge set at 5525 × *g* and 4°C for 30 min to pellet the cell debris. The supernatants were removed, transferred into round-bottom flasks, and evaporated under vacuum to 1 mL using a Büchi rotary evaporator at 55°C. The concentrated 1 mL solutions were carefully transferred into 2 mL microcentrifuge tubes and dried overnight in a heating block set to 55°C. Following the overnight drying step, the evaporated samples were reconstituted in 500 µL of 50% HPLC-grade methanol: milliQ water solvent (1:1, v/v). The samples were vortexed briefly and filtered through 0.22 µm nylon syringe filters into vials fitted with 500 µL inserts. The samples were stored at 4°C until LC–MS analysis.

The cold methanol extraction method described previously has widely been used in metabolomics experiments but does preselect for metabolites extractable in methanol and highly polar/nonpolar compounds may only be partially extracted or be completely lost in the case of very volatile compounds. However, a previous literature has shown that the described method is able to recover most of the secondary metabolites present within the tomato metabolome with high levels of reproducibility (Gómez-Romero et al., 2010; Roldan et al., 2014; Zeiss et al., 2018; Zeiss et al., 2019).

### 2.11 Ultra-high performance liquid chromatography-high-definition mass spectrometry

The samples were analyzed on an UHPLC-quadrupole time-of-flight high-definition MS (UHPLC-qTOF HD-MS) system equipped with an electrospray ionization (ESI) source. The analytes were separated on an Acquity Classic UHPLC system, binary solvent and fixed loop (BSM-FL) configuration. An Acquity HSS T3 reverse-phase column (2.1 × 150 mm × 1.7 µm; Waters Corporation, Milford, MA, United States) was used for chromatographic separation. A binary solvent system consisting of acetonitrile (Romil Chemistry, Cambridge, United Kingdom): milliQ water, with both solvents containing 0.1% formic acid (FA, Sigma-Aldrich, Munich, Germany) and 2.5% isopropanol (Sigma-Aldrich, Munich, Germany), was used. A binary gradient elution method was used over a 30 min run with a flow rate set to 400 μL/min. Three pooled quality control (QC) samples composed of aliquots from all the sample groups, as well as 50% HPLC-grade methanol blanks, were included in the sample list. The QC samples were added to monitor sample stability and feature legitimacy, assess intensity drifts that occur during the data acquisition process, and monitor instrumental efficiency and robustness. Each of the biological samples (originating from three biological replicates) was analyzed in triplicate (analytical repeats) on the UHPLC-MS instrument to improve the precision and accuracy.

2 μL of each sample was injected with the elution starting at 2% (v/v) acetonitrile from 0 to 1 min, raised to 70% acetonitrile from 1 to 22 min, taken up to 95% from 22 to 23 min, and then kept constant at 95% acetonitrile from 23 to 26 min. The composition of the mobile phase was then reverted to 2% acetonitrile from 26 to 27 min, for column cleaning and equilibration from 27 to 30 min. The metabolites present in the extracted samples were chromatographically separated and detected with a high-definition mass spectrometer (Synapt G1, Waters Corporation, Milford, MA, United States), set to acquire accurate data in both positive and negative ionization modes.

The MS conditions were as follows: capillary voltage of 2.5 kV, sample cone voltage of 30 V, microchannel plate detector voltage of 1,600 V, desolvation temperature of 450°C, source temperature of 120°C, cone gas flow of 50 L/h, desolvation gas flow of 550 L/h, *m/z* range of 50–1,500, scan time of 0.2 s, interscan delay of 0.02 s, and the data were acquired in centroid format. The lockmass flow rate was 100 μL/min, with leucine encephalin as a double-point lock spray internal reference solution (50 pg/mL, [M + H]+ = 556.2771 and [M—H]^−^ = 554.2615), continuously sampled every 15 s, producing an average intensity of 350 counts/scan in the centroid mode, with typical mass accuracies between 3 and 5 mDa and a mass accuracy window of 0.5 Da. High-purity nitrogen was used as desolvation, cone, and collision gas. The MS analyses were set up to perform five sequential full scan methods with increasing collision energies ranging from 10 to 50 eV. The method described previously enables the simulation of MS^E^ acquisition, while additionally allowing for the monitoring of individual collision energy channels. This feature greatly facilitates the downstream structural elucidation of compounds. By acquiring data at various collision energies, the method ensures the fragmentation of a diverse range of compounds with varying physicochemical properties.

### 2.12 Data analysis: pre-treatment, pre-processing, and multivariate statistics methods

The data obtained from the UHPLC-MS system were analyzed with the MassLynx™ software (Waters Corporation, Manchester, United Kingdom), with the addition of other statistical programs for multivariate data analysis (MVDA). Pre-treatment and pre-processing steps were according to Tugizimana et al. (2016). Briefly, the raw data were processed with MarkerLynx XS™ 4.2 application manager, with the following parameters: 0.60–21 min retention time (Rt) range of the chromatograms and *m/z* domain of mass range 50–1,500 Da. The Rts were allowed to differ by ± 0.20 min and the *m/z* values by ± 0.05 Da. The mass tolerance was 0.01 Da, and the intensity threshold was 10 counts. Only data matrices with noise level less than 50% (MarkerLynx cut-off) were retained for downstream data analyses. The MarkerLynx application uses the patented ApexPeakTrack algorithm to perform accurate peak detection and alignment. Furthermore, MarkerLynx performs sample normalization based on total ion intensities of each defined peak. Prior to calculating intensities, Savitzky–Golay smoothing and integration was performed (Zhou et al., 2012; Chen et al., 2013; Tugizimana et al., 2016). The generated data matrices were imported into soft independent modelling of class analogy (SIMCA) software, version 14.0 software with the “Omics” skin (Sartorius Stedim Data Analytics AB, Umeå, Sweden) for statistical analyses. Prior to chemometric modelling, the independent variables, i.e.*,* features, within the data matrix were *Pareto*-scaled as a method of data normalization, to accommodate and adjust for measurement errors between minor and major peaks. A non-linear iterative partial least squares algorithm (NIPLS, in-built within SIMCA software) was applied to manage the missing values, with a default threshold of 50% and a correction factor of 3.0. Two unsupervised multivariate methods, namely, principal component analysis (PCA) and hierarchical cluster analysis (HiCA), were applied. PCA is an unsupervised projection-based modelling tool that permits the exploration of the dataset, conclusively revealing the systematic variation present within the variables (Trygg et al., 2007). PCA converts all the correlated variables, i.e., the metabolite feature with their respective intensities, into a smaller number of new uncorrelated variables, described as principal components (PCs) that can be projected onto a lower dimensional space while retaining most of the information embedded in the original dataset (Goodacre et al., 2007; Saccenti et al., 2014; Ren et al., 2015). HiCA is a general algorithmic approach used in cluster analysis, where the observations from the PCA are grouped into clusters based on their varying degrees of (dis)similarity.

In addition, a supervised statistical method, orthogonal projection to latent structures discriminant analysis (OPLS-DA), was used for discriminant analysis and identification of features contributing to differences in the metabolomes. OPLS-DA is typically applied as a supervised method of diagnosing selective differences between the two groups. In this experiment, the OPLS-DA modelling was used to compare the LPS_*R. sol.*_-treated samples to the MgSO_4_ control samples for each incubation interval and to subsequently identify which metabolite features demonstrate the largest impact (i.e., discriminatory power), on class separation between the two analyzed groups (Tugizimana et al., 2013). Without rigorous validation, OPLS-DA modelling might be able to produce unreliable class separation leading to statistically insignificant conclusions (Worley and Powers, 2016). Several methods of validating the OPLS-DA models were consistently applied, e.g., the receiver operating characteristic (ROC) plot construction, response permutation testing, and a seven-fold cross-validation (CV) method (Eriksson et al., 2008). Only models deemed statistically valid were examined and used in data mining process and marker discovery. The variable importance in projection (VIP) plots were generated to assess the calculated importance of the discriminant features that are discovered during the negative control vs*.* LPS_*R. sol.*_ treatment supervised methodologies. The VIP plots in the study had two purposes: first, they acted as a guide to aid in the selection of statistically significant features with VIP scores above a universal threshold of ≥2, and second, they served as a validation method to prevent any potential bias during the feature selection process. The study found that utilizing these plots helped to identify relevant features and minimized the risk of introducing bias during the selection process (Tugizimana et al., 2016).

### 2.13 Metabolite annotation and documentation

The chemical and structural identities of the metabolite features deemed statistically significant/discriminant were elucidated using several parameters that have been outlined (Sumner et al., 2007; Alseekh et al., 2021), including MS spectral-based metabolite identification performance based on sufficient and accurate mass fragment information, accurate calculation of each feature’s elemental composition below 5 mDa error, monitoring the double bond equivalent (DBE) values, and database searches for possible metabolite annotation. A built-in MarkerLynx XS software tool, MassFragment, was used to match possible structures to the observed fragment ions of each feature using novel algorithms. The putative empirical formula of each statistically discriminant feature was searched in the following databases for possible compound matches: ChemSpider <www.chemspider.com>, Dictionary of Natural Products <www.dnp.chemnetbase.com/>, PubChem <http://pubchem.ncbi.nlm.nih.gov/>, METLIN <http://metlin.scripps.edu/>, KEGG Compound database <https:// www.genome.jp/kegg/compound/>, and MetaCyc < https://metacyc.org/>. All metabolites reported were annotated (tentatively identified) according to level 2 of the Metabolomics Standard Initiative (MSI) (Sumner et al., 2007; Spicer et al., 2017). Semi-quantitative analysis of discriminate metabolites, based on relative peak intensities, was performed within SIMCA ver. 14 software with a univariate statistical analysis as indicated in the figure legends.

## 3 Results

**FIGURE 1:**
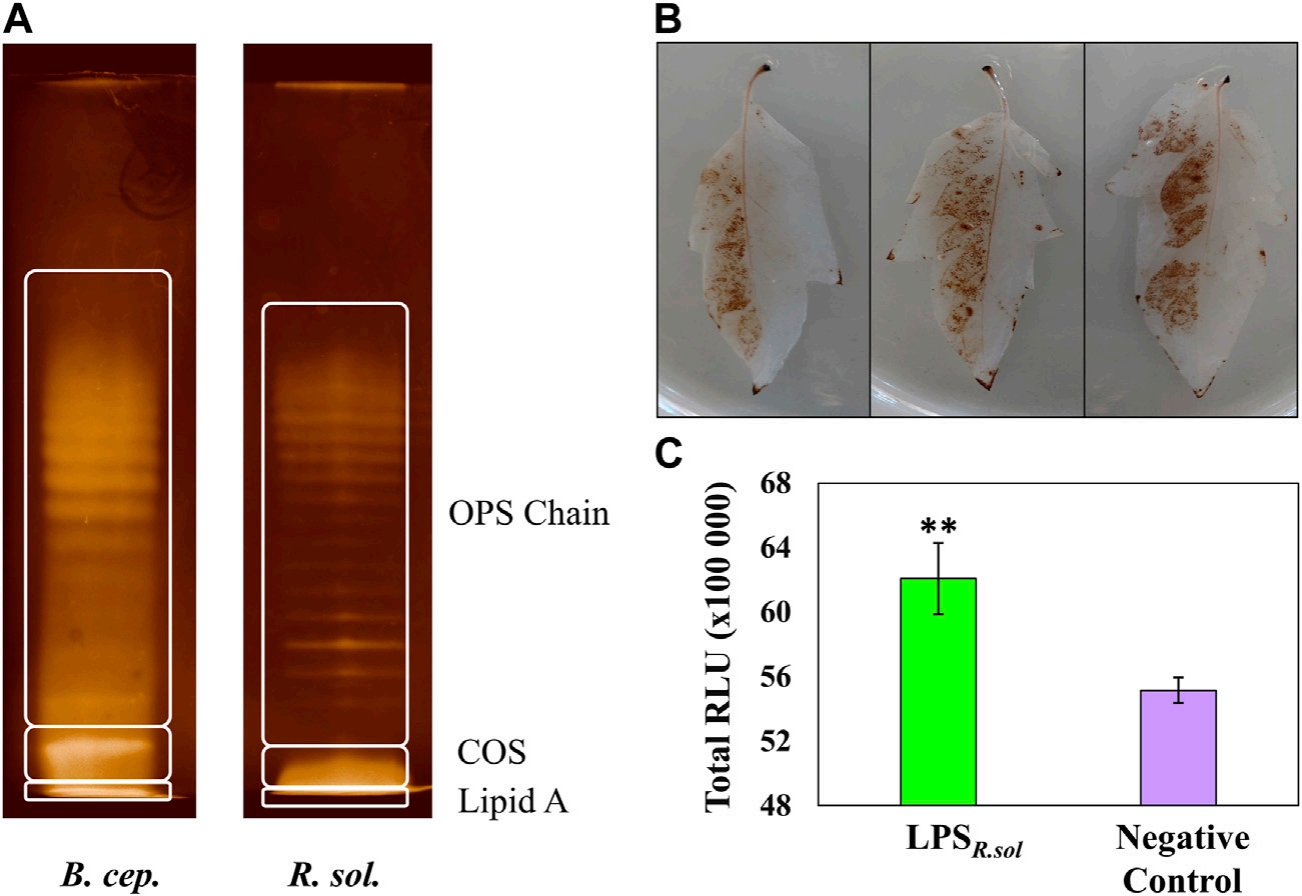
Comparative electrophoretic patterns of LPS from *Ralstonia solanacearum* compared to that of *Burkholderia cepacia* reference **(A)**, and generation of free radical species in the leaf tissue of S. *lycopersicum* in response to LPS_*R. sol.*_ (100 µg/mL) treatment **(B, C)**. LPS banding pattern was visualized using the silver staining method, and the regions indicate the OPS chain, core oligosaccharide, and lipid A. **(B)** Generation of H_2_O_2_ visualized using the peroxidase-dependent 3,3-diaminobenzidine (DAB) stain after 24 h incubation. The left abaxial half of the leaves was pressure-infiltrated with LPS_*R. sol.*_ while the right half served as 8 mM MgSO_4_/water control. **(C)** Total ROS production measured over a 60-minute interval after elicitor inoculation using the horseradish peroxidase-dependent luminescence assay. A pairwise Student’s *t*-test was performed to determine statistical significance between the LPS treatment (green) and negative control (purple), where asterisks indicate the degree of statistical significance (** = *p* ≤ 0.001). Both experiments were replicated (DAB, *n* = 3 and luminescence *n* = 24), as three independent experiments.

LPS from Gram-negative phytopathogens, including *R. solanacearum,* have been reported to be capable of triggering immune responses in several plant species (Esposito et al., 2008; Li et al., 2014). The LPS_*R. sol*._ was purified (Figure 1A), chemically characterized, and structurally elucidated. The metabolomics study was subsequently designed to: (i) discover whether an untargeted approach was capable of capturing the subtle LPS_*R. sol.*_-induced perturbations within the leaf metabolome of tomato plants and (ii) determine which phytochemical classes and metabolic pathways were positively correlated to the overall host immune/defense response following LPS_*R. sol.*_ perception.

### 3.1 LPS-induced oxidative burst

Prior to metabolomics analysis, several assays were performed to confirm that the tomato cultivar was capable of perceiving the LPS_*R. sol.*_ elicitor. The DAB staining method was performed to detect the production of hydrogen peroxide (H_2_O_2_) and other associated free radical species in the LPS_*R. sol.*_ -inoculated leaf tissues, suggestive of initial host perception (Flores-Cruz and Allen, 2009). The left abaxial side of the leaves was treated with the LPS_*R. sol.*_ elicitor, while the right side was treated with a negative control (Figure 1B). The localized production of the brown precipitate validated the host plant’s capacity to perceive the LPS_*R. sol.*_, triggering the oxidative burst, leading to free radical production (Figure 1C). A more sensitive luminescence assay was performed to corroborate the findings observed during the histochemical stain procedure. The total LPS_*R. sol.*_-induced reactive oxygen species (ROS) production was measured and compared to a negative control supplemented with water (Figure 1C). The overall luminescence results (Figures 1B, C) confirmed the host plant’s ability to respond to the LPS_*R. sol.*_ elicitor through the production of ROS, albeit at a lower metabolic level compared to flagellin-derived peptides flg22 and flg28 (Zeiss et al., 2021a).

### 3.2 Electrophoretic analysis of LPS from *Ralstonia solanacearum*

Electrophoretic analyses (SDS-PAGE gels) were used to: i) analyze the purity of the extracted LPS_*R. sol.*_, ii) determine the dominant LPS type extracted from the bacterial pathogen, and iii) assess whether the observed LPS_*R. sol.*_ banding pattern corresponded to what has been previously described in the literature (Yang et al., 2013). The LPS of *B. cepacia* (LPS_*B.cep.*_) has been well-described in the literature (Gerber et al., 2004) and was included alongside the LPS_*R. sol.*_ as a comparative reference. The LPS components were revealed as gel bands using the sensitive silver staining method. The banding patterns revealed that both the LPS_*R. sol.*_ and LPS_*B.cep.*_ contain key structural features (Figure 1A), i.e., both containing the lipid A moiety, the COS, and the variable OPS chain (Erridge et al., 2002). Overall, the banding pattern results indicate that *R. solanacearum* has S-LPS, i.e., it contains the three structural components of LPS (Li et al., 2014). Since the exact type of the LPS and the structural features of its constituent substructures can determine its immungenic properties (Madala et al., 2012), structural analysis of the purified LPS was undertaken.

### 3.3 Structural characterization of the lipid A and OPS fractions of LPS from *Ralstonia solanacearum*

Evaluation of the saccharide composition and absolute configuration was conducted on intact LPS_*R. sol*__*.*_ Compositional analysis revealed the occurrence of predominant sugar species consistent with the presence of an OPS moiety. LPS_*R. sol.*_ in fact displayed mainly L-rhamnose (L-Rha), 2-amino-2-deoxy-D-glucose (D-GlcNAc), and L-xylose (L-Xyl), and in minor amounts 4-amino-4-deoxy-L-arabinose, D-glucose, L-*glycero*-D-*manno*-heptose, and 3-deoxy-D-*manno*-oct-2-ulopyranosonic acid (Kdo). Linkage analysis revealed mainly terminal Xyl, 2-substituted, 3-substituted Rha, 3-substituted GlcNAc, and also in minor amounts, 3,4-disubstituted Rha. This analysis also showed that all residues were in the pyranose form.

Fatty acid analysis disclosed the occurrence of (*R*)-3-hydroxytetradecanoic acid [C14:0(3-OH)], (*S*)-2-hydroxytetradecanoic acid [C14:0(2-OH)], (*R*)-3-hydroxyhexadecanoic acid [C16:0(3-OH)], (*S*)-2-hydroxyhexadecanoic acid [C16:0(2-OH)], dodecanoic (C12:0), tetradecanoic (14:0), and hexadecanoic (16:0) acids, which was consistent with the lipid A fatty acid composition previously described for *Ralstonia* LPS (Varbanets et al., 2003; Zhang-Sun et al., 2019).

In order to establish the chemical structure of the OPS and lipid A, a mild acid hydrolysis was performed to yield the water-soluble OPS fraction in the liquid phase and the insoluble lipid A portion as the precipitate. The OPS was then further purified by size exclusion chromatography and inspected by means of 1D and 2D NMR spectroscopy. A set of 2D homo- and hetero-nuclear NMR spectra were recorded to establish all the spin systems and to characterize the OPS sequence. The assignment of each spin system was accomplished by tracing the spin connectivity as observed in DQF-COSY and TOCSY spectra. The recognition of each carbon atom was achieved by analyzing the one-bond heteronuclear correlations visible in the HSQC spectrum. The anomeric configuration of each monosaccharide was established by evaluating the intra-residue NOE correlations, which were identified in the NOESY spectrum, and the ^3^ *J*_H-1,H-2_ coupling constants attained from the DQF-COSY spectrum. Vicinal ^3^ *J*_H,H_ coupling constants allowed the assignment of the relative configuration of each sugar unit. Finally, the whole OPS-repeating unit sequence was obtained by merging data from both the inter-residue NOE contacts and the heteronuclear long-range correlations visible in the HMBC spectrum.

**FIGURE 2:**
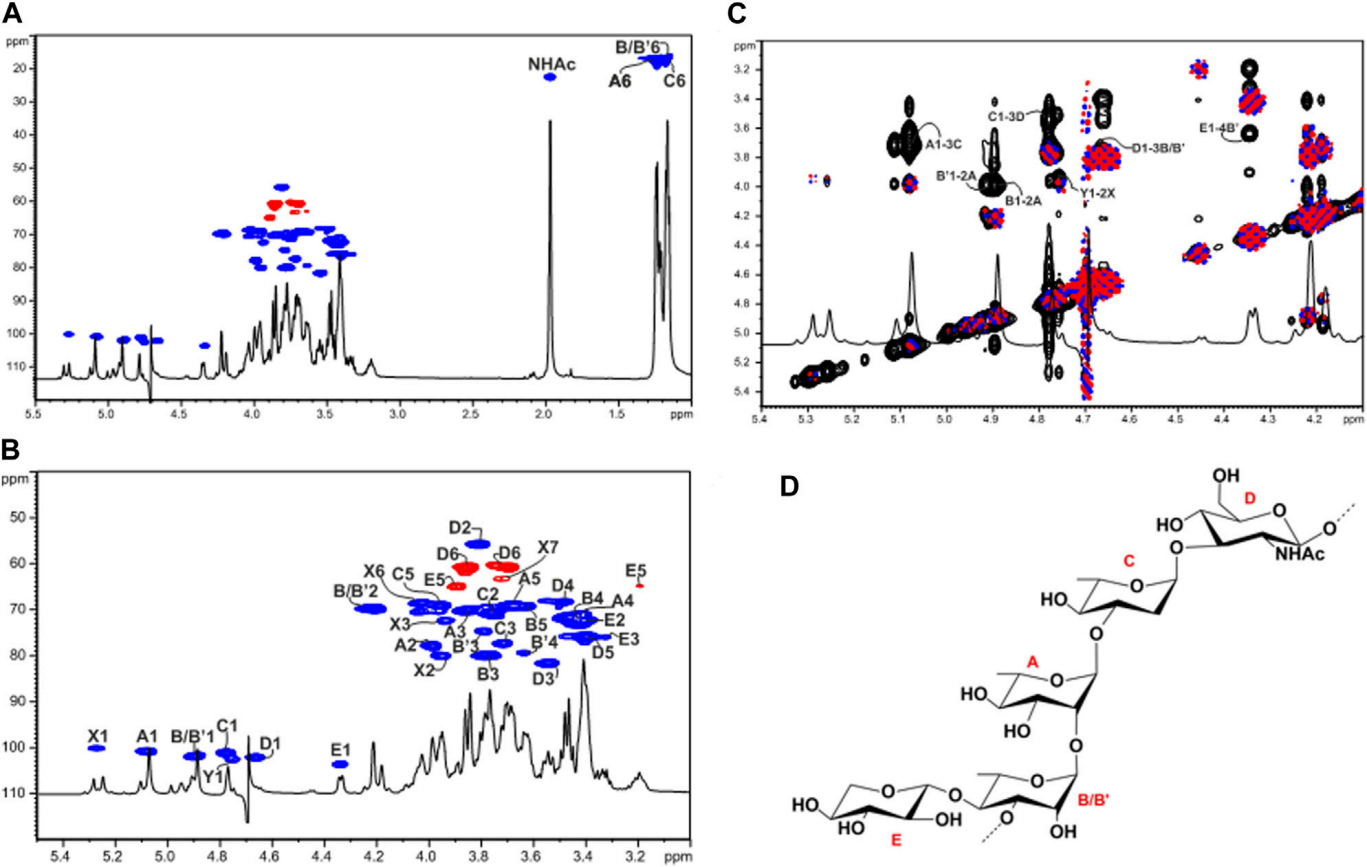
Structural characterization of the O-polysaccharide (OPS) moiety from LPS_*R. sol.*_ **(A)** Overlap of the full ^1^H and ^1^H,^13^C HSQC spectra of the OPS from LPS_*R. sol*._ after mild acid treatment. **(B)** Zoom of the overlapped ^1^H and ^1^H,^13^C HSQC spectra where the main one-bond heteronuclear correlations are indicated. Numbering of sugar residues is as reported in Supplementary Table S1. **(C)** Zoom of the overlapped ^1^H, COSY (blue and pink) and NOESY (black) spectra. The key inter-residue NOE correlations involving sugar moieties are indicated; letters are as in Supplementary Table S1. **(D)** Structure of the elucidated OPS pentasaccharidic repeating unit of the LPS_*R. sol*._ The main tetrasaccharide OPS repeating unit is devoid of the xylose residue.

In the ^1^H NMR spectrum of the OPS (Figure 2), the anomeric region showed five main proton signals that were assigned to five different spin systems (**A**–**E**, Supplementary Table S1, Figures 2A, B). All sugar residues were present as pyranose rings according to ^13^C chemical shift values (Figure 2, Supplementary Table S1) and in agreement with the linkage analysis.

Spin systems **A** (H-1 at 5.07 ppm), **B** (H-1 at 4.89 ppm), and **C** (H-1 at 4.77 ppm) displayed correlations in the TOCSY spectrum with methyl signals at *δ*_H_ = 1.25, 1.18, and 1.16 ppm, respectively, and were assigned to α-rhamnose residues. The *manno* configuration was established by analyzing ^3^ *J*_H,H_ coupling constant values, while the α-anomeric configuration was assigned by the ^1^ *J*_CH_ coupling constant value of 175.8 Hz and further confirmed by the chemical shifts of *δ* 69.3–69.1 ppm for C-5 of **A**, **B**, and **C** (Lipkind et al., 1988).

Spin system **D** (H-1 at 4.66 ppm) was attributed to a β-GlcNAc as proven by the H-2 proton that showed a correlation with a nitrogen-bearing carbon atom at 55.8 ppm (Supplementary Table S1, Figure 2B). The chemical shifts of ring protons that agreed with the *gluco*-configuration of pyranose rings as well as the NOE correlations between H-1, H-3, and H-5, and the large ^3^ *J*_H-1,H-2_ coupling constant value. Residue **E** (H-1 at 4.34 ppm) was assigned to a β-Xyl as judged by the ^3^ *J*_H-1,H-2_ coupling constant value of 8.1 Hz, the chemical shifts of ring protons typical of *gluco*-configured pyranose rings, as well as by the observation of H-5 and C-5 resonating at 3.90/3.19 and 65.0 ppm, respectively.

The down-field shifts of carbon signals identified glycosylated positions at O-2 of **A** and at O-3 of **B**, **C**, and **D**, while residue **E** was identified as a terminal unit. The NOESY spectrum of the OPS showed strong inter-residue correlations between the following protons: H-1 of Rha **A** with H-3 of Rha **C** at *δ* 5.07/3.72, H-1 of Rha **C** with H-3 of GlcNAc **D** at *δ* 4.77/3.53, H-1 of GlcNAc **D** with H-3 of Rha **B** at *δ* 4.66/3.77, and H-1 of Rha **B** with H-2 of Rha **A** at 4.89/3.99 (Figure 2C). Therefore, this analysis led to the identification of the linear tetrasaccharidic OPS repeating unit:

[→3)-α-L-Rha-(1→3)-β-D-GlcNAc-(1→3)-α-L-Rha-(1→2)-α-L-Rha-(1→].

As for the terminal Xyl **E**, a strong inter-residue NOE correlation between its anomeric proton at 4.34 ppm and a ring proton resonating at 3.63 ppm (*δ*_C_ at 79.4 ppm, Supplementary Table S1, Figure 2C), which was assigned to H-4 of an additional α-Rha labelled **B’** (H-1 at 4.90 ppm), allowed the determination of a minor xylosylated branched substructure where residue **B** was not stoichiometrically substituted at its position 4 by β-Xyl **E** (Figure 2D).

Finally, two spin systems labelled as **X** and **Y** were also identified and assigned as constituents of the COS, i.e., 2,3-α-heptose (**X**) and a terminal α-Rha (**Y**) that is linked at its position O-2, as previously described (Zdorovenko et al., 2008).

**FIGURE 3:**
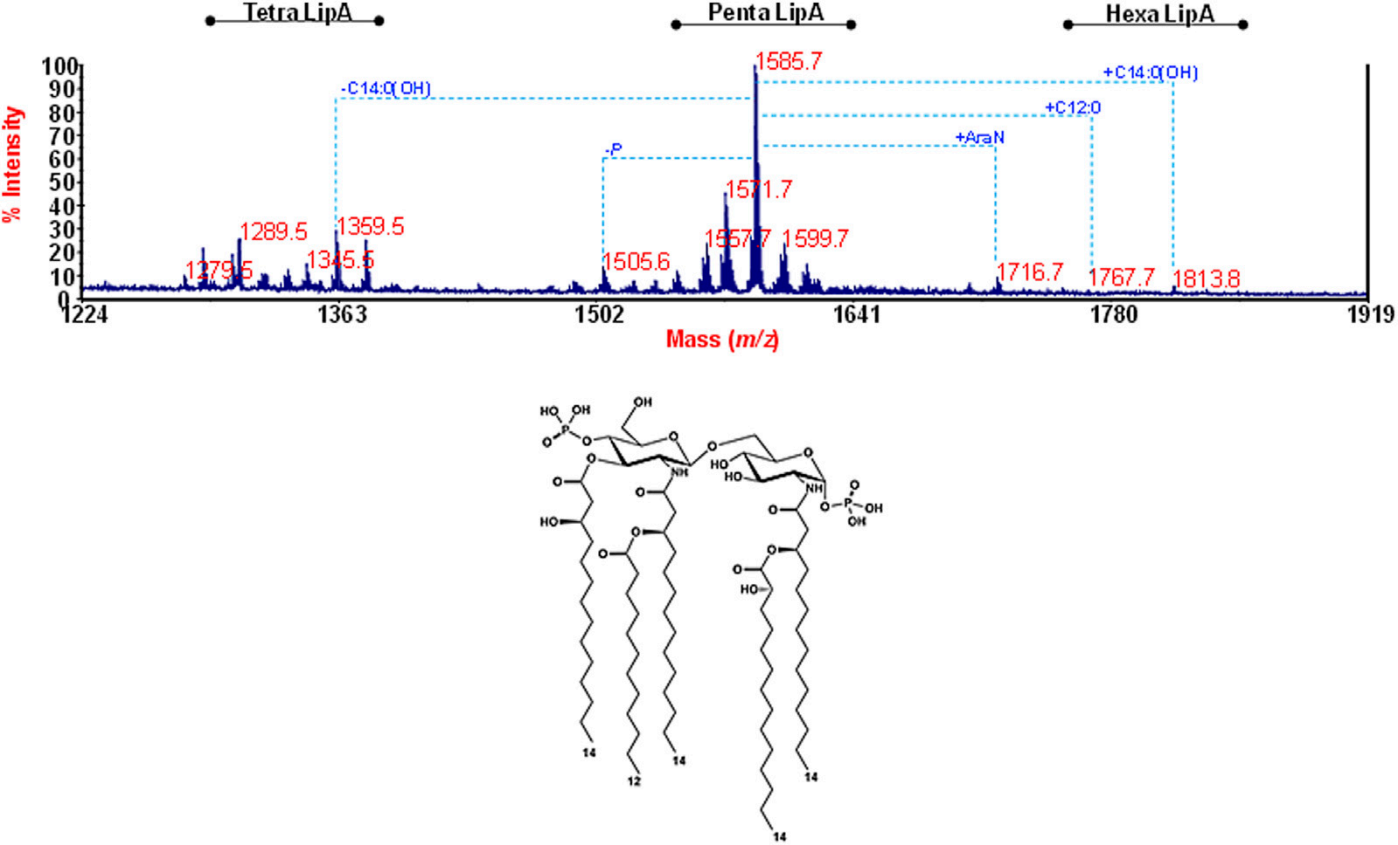
Structural determination of the lipid A fraction. Reflectron MALDI-TOF mass spectrum, recorded in negative polarity of lipid A from *R. solanacearum* obtained after acetate buffer treatment of LPS_*R. sol*_. The lipid A species are labelled as tetra-, penta-, and hexa lipid A indicating the degree of acylation. The proposed structure for the main lipid A species detected at *m/z* 1,585.7 is reported in the inset.

As for the lipid A moiety, this was investigated by negative-ion MALDI-TOF MS and MS/MS. The reflectron MALDI-TOF MS spectrum is reported in Figure 3, and it showed the occurrence of a predominant cluster of peaks that was assigned to [M-H]^–^ *bis*-phosphorylated penta-acylated lipid A species with the main peak at *m/z* 1,585.7 matching with a lipid A carrying two phosphate groups, four 14:0(OH) and one 12:0. Of note, minor species at *m/z* 1,716.7, m*/z* 1,767.7, and *m/z* 1,813.8 were detected and attributed to lipid A additionally carrying Ara4N, 12:0 or 14:0(OH), respectively. Tetra-acylated lipid A species were also identified at about *m/z* 1,359.5. In order to locate the acyl chains with the respect to the glucosamine disaccharide backbone, a negative ion MALDI-TOF MS/MS analysis was conducted on several peaks and revealed a 3 + 2 symmetry of the fatty acids for all penta-acylated species. As an example, the analysis of the MS/MS spectrum of the precursor ion at *m/z* 1505.6 (Supplementary Figure S1), representative of *mono*-phoshorylated penta-acylated lipid A species, highlighted that the non-reducing glucosamine carries the phosphate, two primary 14:0(3-OH) and one secondary 12:0, while the reducing glucosamine bears one primary *N*-linked 14:0(3-OH) in turn acylated by a secondary 14:0(2-OH). These results were consistent with previous data about *Ralstonia* lipid A (Varbanets, et al., 2003; Zhang-Sun et al., 2019), although in this case, a major hypo-acylation degree was noticed.

### 3.4 Reverse-phase chromatography separation of leaf extracts with mass spectrometric detection

**FIGURE 4:**
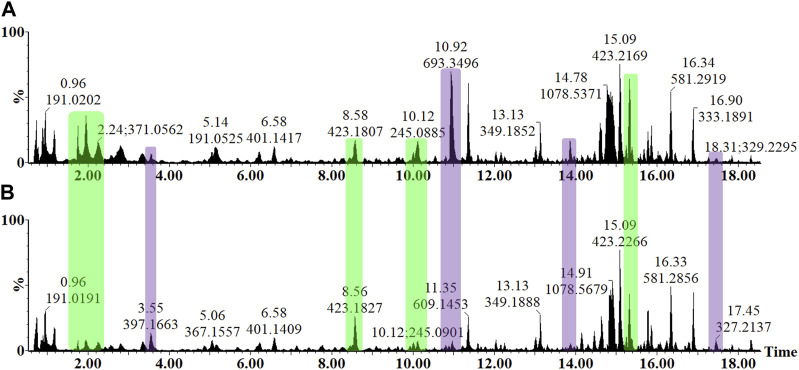
Overlaid UHPLC-MS BPI chromatograms (ESI^−^) of methanolic leaf extracts from the LPS_*R. sol.*_ elicitor-treated tomato plants. A comparison of the metabolite profiles at the 16 h after infiltration time interval **(A)** MgSO_4_ negative control and **(B)** LPS treatment revealed concentration-linked variation in relative peak intensities. The *y*-axes of the two chromatograms are linked and represent the relative abundance (%) of the metabolite features at their respective retention times (min). The changes in peak intensities (green) and/or the presence/absence of peaks (purple) could be observed, reflecting the LPS_*R. sol.*_-induced perturbation of leaf metabolism.

In the untargeted UHPLC-MS workflow, the LC dimension was used for the adequate separation and resolution of the various phytochemicals within the crude sample extracts, while the MS component was used for the detection and downstream structural elucidation of these small molecules. As a dedicated tool in metabolomics, UHPLC-MS workflow is especially suitable to capture the phytochemical diversity found in plants, which includes the semi-polar species of secondary metabolites that have defensive or protective functions. The metabolite profiles of the LPS_*R. sol.*_ treatment and control tomato leaf samples are visually presented as an *y*-axis linked overlay of two base peak intensity (BPI) chromatograms (Figure 4). The chromatographic data generated in negative electrospray ionization (ESI) mode are presented due to the various metabolite classes exhibiting an enhanced level of ionization in this mode. The chromatograms demonstrate the level of resolution required for phytochemical analyses and highlight the underlying changes in the metabolism influenced by the LPS_*R. sol.*_-treatment (Figure 4). Qualitative variance is reflected by peak intensity, where the *y*-axis indicates the relative peak intensity each metabolite at their respective Rts (min). The presence/absence of peaks, as well as quantitative differences, again highlights the effect of the LPS_*R. sol*_-treatment on the leaf metabolome of *S. lycopersicum*. Comparative figures of treatment vs. control at the 24 and 32 h timepoints are provided as Supplementary Figures S2, S3, while Supplementary Figure S4 provides a time dependent comparison of the extracts at 16, 24, and 32 h post treatment.

### 3.5 Multivariate statistical analysis and modelling

**FIGURE 5:**
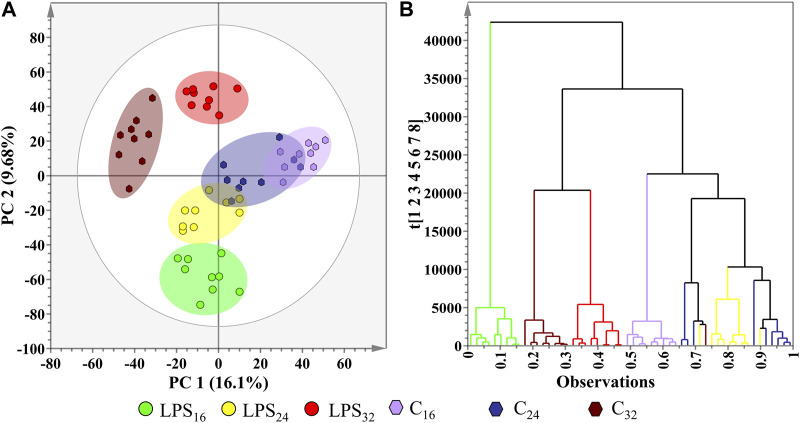
Investigation of the underlying trends in the *S. lycopersicum* leaf metabolome data matrices after treatment with LPS_*R. sol.*_ with the application of two unsupervised learning methods. **(A)** 2D PCA scores plot illustrating the formation of observed group clusters at the allocated after treatment incubation time points (16, 24, and 32 h), with the addition of a corresponding negative controls (C). The generated PCA model yielded a R^2^X(cum) value of 60.2% and a Q^2^(cum) value of 35%. The ellipse on the scores plot represents Hotelling’s T^2^ confidence interval at 95%. **(B)** Ward-linkage HiCA dendrogram corresponding to cluster points plotted in (**A**), showing the hierarchical outline of the negative controls and LPS_*R. sol.*_-treatment time points.

Following UHPLC-MS analysis and data pre-processing, the data matrices were subjected to multivariate data analytic procedures to sort through the data, revealing the underlying trends and patterns that may have been hidden during the LC step. The aforementioned trends equate to fluctuations in the leaf metabolic profiles across the time intervals in response to LPS_*R. sol.*_ treatment. The multidimensional dataset obtained was subjected to PCA, an unsupervised projection-based modelling tool that permits the exploration of the dataset, conclusively revealing the systematic variation present within the variables (Trygg et al., 2007). PCA converts all the correlated variables into a smaller number of principal components that are projected onto a lower dimensional space while retaining most of the information embedded in the original datasets (Goodacre et al., 2007; Saccenti et al., 2014; Ren et al., 2015). A desirable outcome for PCA is a scores plot where the groups examined produce statistically distinct clusters (Worley and Powers, 2013). Unless predetermined by the experimental design, which is not applicable in this instance, the absence of group separation would indicate a failed result. Examining the PCA scores plot (Figure 5A), it can be seen that the first two principal components (PC1 vs*.* PC2) only explained a quarter of the total variation within the dataset. The decreased level of explained variation can be attributed to the host treatment with a single elicitor, *i.e.*, LPS_*R. sol.*_, rather than a cocktail of MAMPs, which is frequently observed in pathogen infection studies (Zeiss et al., 2019). The scores plot revealed the clear group clustering of the conditions moving in a convergent manner as the incubation time points continue.

A partial overlap between some of the scores plot clusters was observed (Figure 5A), suggestive of metabolic similarities in the groups whose presence or cellular concentration have remained unaltered in response to the LPS_*R. sol.*_ treatment. This pattern implies that not all metabolic pathways present in the metabolome were influenced by LPS_*R. sol.*_ treatment. Again, this observation is consistent with a single elicitor treatment rather than a cocktail of MAMPs (Zeiss et al., 2019; Zeiss et al., 2021a). When superimposed with the Hotellings T^2^ 95% confidence interval ellipse, no outliers were observed on the PCA scores plot. In addition to PCA, the dataset was subjected to HiCA (Figure 5B), as a complimentary unsupervised method of data exploration that constructs a hierarchy of the score clusters using a dendrogram to reveal trends that may be hidden during PCA. The HiCA revealed that the greatest variation in metabolite profiles was observed between the control and treated groups during the initial 16 h incubation point. A possible overlap between the metabolite profiles of the control and treated groups was also observed during the 24 and 32 h incubation points, respectively. This observation is suggestive of the host’s metabolism recovering from the initial perturbation and subsequent defense response to rapidly re-establish a new homeostatic level.

**FIGURE 6:**
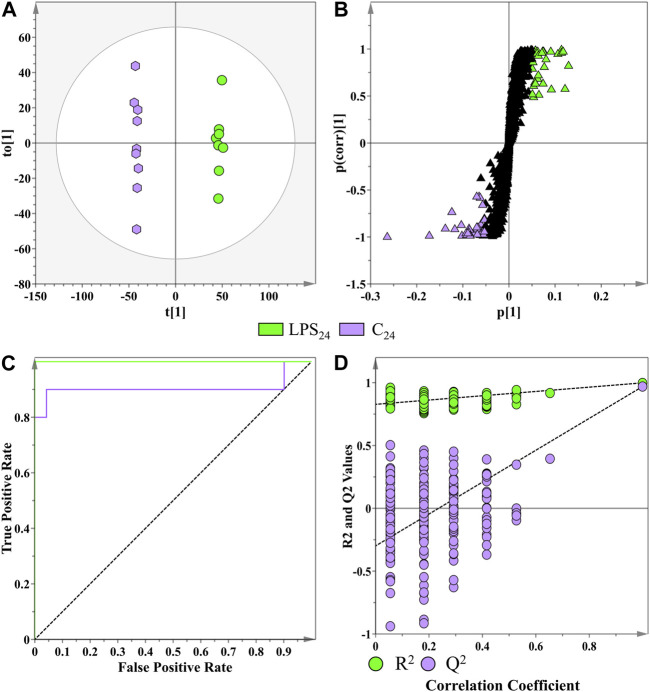
Application of a supervised learning method for the data processing of extracts from *S. lycopersicum* leaf tissue (LPS_*R. sol.*_ treatments and MgSO_4_ negative controls) at the 16 h incubation time point. **(A)** OPLS-DA scores plot showing the group separation of control vs. treated (LPS_*R. sol.*_, green vs. controls, purple) conditions. The calculated model yielded R^2^X (cum) = 40.6%, R^2^Y (cum) = 99.8%, and Q^2^ (cum) = 97%. Model validation by seven-fold CV-ANOVA displayed a level of statistical significance with *p* value = 5.337 x 10^−9^. **(B)** Corresponding OPLS-DA loading S-plot. Relevant variables far out in the loadings S-plot (*x, y* ≥ 0.05, 0.5) were selected and represented potential discriminating variables. **(C)** Receiver operating characteristic (ROC) curve summarizing the selective ability of a binary classifier (S-plot), with a classifier having a perfect discrimination producing a ROC curve that passes through the top left corner to indicate 100% sensitivity and specificity. **(D)** Response permutation test plot (*n* = 200) for the OPLS-DA model.

As a supervised method of binary class separation, OPLS-discriminant analysis was applied to find a linear relationship between the multivariate predictor matrix (e.g., the spectrometric data of the biological samples) and the response matrix (e.g., the MgSO_4_ control and LPS_*R. sol.*_-treated samples) (Triba et al., 2014). A representative OPLS-DA scores plot, the S-plot for feature selection, and the associated model validations are presented in Figures 6A–D. The OPLS-DA scores plot (Figure 6A) displayed clear class separation between the control and the LPS_*R. sol.*_ sample groups incubated at the 24 h time interval. The information relating to the other computed supervised models are presented in Supplementary Table S2. The corresponding OPLS-DA loadings S-plot (Figure 6B) highlights the features (*m/z* ions) deemed statistically important that are positively/negatively correlated to the LPS_*R. sol.*_ treatment at the selected incubation time points. Features situated at the extremes of the S-plot have a combination of high influence and reliability to OPLS-DA and are relevant in the search for positively/negatively correlated metabolite markers. Positively correlated metabolite features with |p(corr)| values of ≥0.5 and covariance values of |(p1)| ≥ 0.05 were selected for further downstream analysis (Trygg et al., 2007). The abovementioned parameters were selected as part of a feedback loop based on the application of descriptive statistics (analysis of variance, ANOVA) on all of the features present in the OPLS-DA S-plot. In the parameter determination process, a statistical cut-off of *p* value ≤0.05 was used for the ANOVA. The lowest p(corr)| and |(p1)| values of features that adhered to the aforementioned cut-off values were used to create the initially described parameters. These parameters are largely data-dependent and thus a data-driven method was implemented to guide the selection of statistically relevant features, while simultaneously excluding the selection of false positives. The quality of each OPLS-DA model was assessed based on the number of components computed for each model, the calculated R^2^X(cum), R^2^Y(cum), and Q^2^(cum) values, as well as the *P*_CV-ANOVA_ value (Supplementary Table S2). The overall reliability and significance of the OPLS-DA models were evaluated using the seven-fold ANOVA testing of cross-validation (CV-ANOVA) diagnostic tool, where supervised models producing *P*_CV-ANOVA_ values of <0.05 were deemed statistically viable (Eriksson et al., 2008). The *P*_CV-ANOVA_ values for each computed OPLS-DA model are provided in Supplementary Table S2.

The performance of the OPLS-DA model, in terms of selectivity as the discrimination threshold is varied, was evaluated by constructing a receiver operating characteristic (ROC) curve (Figure 6C) (Westerhuis et al., 2008). As a binary classifier, the OPLS-DA model displayed perfect discrimination of the LPS_*R. sol.*_ group (Figure 6C). The calculated area under the curve for each model was tabulated in Supplementary Table S2. The predictive capacity of the OPLS-DA models was validated using a response permutation test (*n* = 200—Figure 6D). The permutation test evaluates whether the classification of the individuals in the two designed control and treatment groups is statistically better than a random classification into two arbitrary groups (Westerhuis et al., 2008). The permutation test revealed that the originally computed models produced higher calculated *R* ^2^ and Q^2^ values compared to the 200 model permutations, concluding that the obtained OPLS-DA models were statistically superior to the generated permutations. The values from all the permutated models for all the treatment conditions were *R* ^2^ < 0.97 and Q^2^ < −0.05 (Supplementary Table S2).

### 3.6 Feature selection and metabolite annotation

The experiments focused on features positively correlated to LPS_*R. sol.*_ treatment. This selection was made using the OPLS-DA loading S-plots (Figure 6B) and VIP scores ≥2. The cut-off values of |p(corr)| and |(p1)| previously mentioned were determined based on the application of descriptive statistics (ANOVA), which also included the calculation of control- and treatment-averaged peak intensities, the standard deviation, the coefficient of variation, the fold change between the two groups, and the *p* value on the selected features (Zeiss et al., 2021a). In the parameter determination process, a statistical cut-off of *p* value ≤0.05 was used for ANOVA. The lowest p(corr)| and |(p1)| values of features that adhered to the aforementioned cut-off values were used to create the initially described parameters.

The application of descriptive statistics supplied basic information about the unique features in the control and LPS_*R. sol*_-treated samples and reduced the risk of discovering false positive markers. The integration of descriptive statistics converted the data analysis step from a qualitative exploration (e.g., monitoring the OPLS-DA loadings S-plot) to a semi-quantitative analysis (e.g., comparing relative peak intensities) (Zeiss et al., 2021a). Metabolite features with threshold values above *p* value ≤0.05 and a coefficient of variation ≤30% were excluded from the analysis.

### 3.7 Investigation of discriminant phytochemicals

A total of 32 specialized metabolites positively correlated to the LPS_*R. sol.*_ treatment in the tomato leaf tissue were annotated (Table 1) from 3,479 original variables present in the processed data matrix. From an experimental perspective, the metabolites were annotated based on accurate mass to (i) measure the mass of *m/z* ions to a degree of accuracy and precision and (ii) limit the number of possibilities during the calculation of elemental composition (Brenton and Godfrey, 2010). The use of accurate mass also facilitated the analysis of mass fragmentation patterns of each compound.

**TABLE 1:** Annotation of metabolite signatures from the leaf tissue of *S. lycopersicum* displaying a positive correlation to the *R. solanacearum*-derived lipopolysaccharide treatment at selected time intervals (16, 24, and 32 h). The metabolites were annotated in both electrospray ionization modes as indicated using liquid chromatography coupled to high-definition mass spectrometry (UHPLC-HDMS).

#	Rt (min)	*m/z*	ESI mode	Putative identification^a^	Chemical formula	Mass error (mDa)	Diagnostic ions	Metabolite class
1	1.00	115.002	[M–H]^-^	Fumaric acid	C_4_H_4_O_4_	−0.1	-	Organic acid
2	1.17	133.012	[M–H]^-^	Malic acid	C_4_H_6_O_5_	−2.8	-	Organic acid
4	1.96	166.084	[M + H]^+^	Phenylalanine	C_9_H_11_NO_2_	−2.6	120	Amino acid
3	2.25	371.058	[M–H]^-^	Caffeoyl glucaric acid isomer I^b^	C_15_H_16_O_11_	−4.7	209	Hydroxycinnamic acid derivative
5	2.29	249.122	[M–H]^-^	Caffeoyl putrescine	C_13_H_18_N_2_O_3_	−5.7	411, 178, and 87	Hydroxycinnamic acid amide
6	2.80	371.058	[M–H]^-^	Caffeoyl glucaric acid isomer II	C_15_H_16_O_11_	−6.2	209	Hydroxycinnamic acid derivative
7	3.16	188.069	[M + H]^+^	Indole acrylic acid	C_11_H_9_NO_2_	−2.3	146, 142, and 118	Indole compound
9	3.56	397.167	[M–H]^-^	Benzoyl ornithine glycoside	C_18_H_26_N_2_O_8_	2.4	293 and 235	Benzoic acid derivative
10	4.85	431.154	[M–H]^-^	Benzoyl alcohol dihexose	C_19_H_28_O_11_	−0.8	108	Benzoic acid derivative
11	5.14	353.083	[M–H]^-^	Caffeoyl quinic acid	C_16_H_18_O_9_	−5.1	191, 179, and 135	Chlorogenic acid
12	7.76	351.127	[M–H]^-^	Feruloyl serotonin	C_20_H_20_N_2_O_4_	−9.8	321, 192, and 175	Hydroxycinnamic acid amide
13	8.56	423.184	[M–H]^-^	Diprenyl eriodictyol	C_25_H_28_O_6_	3.3	355 and 287	Flavonoid
14	8.79	367.100	[M–H]^-^	Feruloyl quinic acid	C_17_H_20_O_9_	−8.4	191 and 161	Chlorogenic acid
15	10.05	245.090	[M–H]^-^	Acetyl tryptophan	C_13_H_14_N_2_O_3_	−3.6	203	Amino acid derivative
16	10.50	203.080	[M–H]^-^	Tryptophan	C_11_H_12_N_2_O_3_	−4.8	188 and 159	Amino acid
17	10.93	693.351	[M–H]^-^	*N’,N″,N‴*-tris-(dihydrocaffeoyl)spermine	C_37_H_49_N_4_O_9_	−1.3	531, 457, 293, and 222	Hydroxycinnamic acid amide
18	11.21	609.139	[M–H]^-^	Rutin	C_27_H_30_O_16_	−6.6	463 and 301	Flavonoid
19	12.14	449.164	[M–H]^-^	PA (18:1, ketol)	C_21_H_38_O_8_P	−1.5	295, 279, 169, and 154	Lipid species
20	13.82	282.112	[M–H]^-^	Coumaroyl tyramine	C_17_H_17_NO_3_	−0.1	147	Hydroxycinnamic acid amide
21	13.87	1096.570	[M–H + FA]^-^	Hydroxy tomatine	C_51_H_86_NO_24_	7.9	1,050 and 416	Steroidal glycoalkaloid
22	14.28	312.121	[M–H]^-^	Feruloyl tyramine	C_18_H_19_NO_4_	−3.2	192 and 178	Hydroxycinnamic acid amide
23	14.46	453.231	[M–H]^-^	LPG (14:1)	C_20_H_38_O_9_P	3.7	379, 371, and 299	Lipid species
24	14.68	1078.550	[M–H + FA]^-^	α-Tomatine	C_50_H_83_NO_22_	3.1	578, 528, and 416	Steroidal glycoalkaloid
25	14.78	1065.570	[M–H]^-^	Tomatidine tetrahexoside	C_51_H_86_NO_22_	7.3	416	Steroidal glycoalkaloid
26	15.24	447.219	[M–H]^-^	PA (18:2, ketol)	C_21_H_36_O_8_P	0.5	293, 277, 169, and 154	Lipid species
27	16.41	291.209	[M–H]^-^	OPDA	C_18_H_28_O_3_	−0.8	247 and 222	Jasmonic acid precursor
28	16.79	327.215	[M–H]^-^	TriHODE isomer I	C_18_H_32_O_5_	−3.2	309	Lipid species
29	17.27	335.221	[M–H]^-^	DiHETE	C_20_H_32_O_4_	−6.5	319 and 303	Lipid species
30	17.47	327.215	[M–H]^-^	TriHODE isomer II	C_18_H_32_O_5_	−3.2	309	Lipid species
31	17.49	329.231	[M–H]^-^	TriHOME isomer I	C_18_H_34_O_5_	−3.5	312, 297, and 281	Lipid species
32	17.86	419.219	[M + H]^+^	PA (17:2)	C_20_H_36_O_7_P	0.3	265, 249, 169, and 154	Lipid species
33	18.32	329.230	[M–H]^-^	TriHOME isomer II	C_18_H_34_O_5_	−5.5	312, 297, and 281	Lipid species

^a^ The metabolite features were annotated according to level 2 of the Metabolomics Standards Initiative (Sumner et al., 2007).

^b^ Geometrical or positional isomers that eluted at different Rts are indicated as isomer I and isomer II, respectively.

*** Abbreviations: DiHETE, dihydroxy eicosatetraenoic acid; FA, formic acid adduct (46 Da), LPG, monoacylglycerophosphoglycerol; OPDA, oxo-phytodienoic acid; PA, phosphatidic acid; TriHODE, trihydroxy octadecadienoic acid; and TriHOME, trihydroxy octadecenoic acid.

**FIGURE 7:**
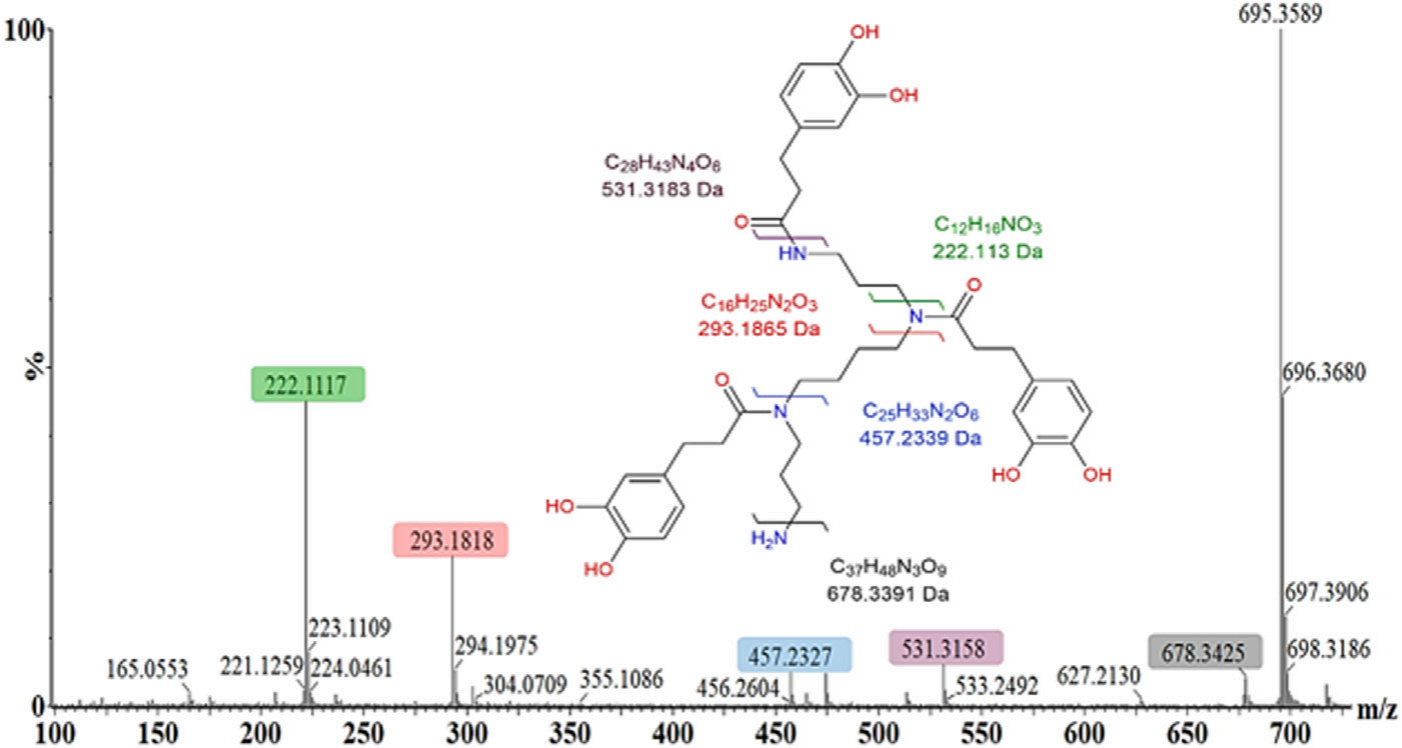
Mass fragmentation pattern of *N′,N″,N”’*-tris(dihydrocaffeoyl)spermine (*m/z* 695.350) in the positive ESI mode. MassFragment software facilitated structural elucidation and compound identification, using the spectral patterns in both ionization modes (ESI negative mode not shown). The molecular ion is 695.359 [M + H]^+^, while the main fragment ions observed are 531.316 [M + H-C_9_H_8_O_3_]^+^, 457.233 [M + H-C_12_H_18_N_2_O_3_]^+^, 293.182 [M + H-C_21_H_26_N_2_O_6_]^+^, and 222.112 [M + H-C_25_H_35_N_3_O_6_]^+^.

The steps associated with the general structural elucidation and metabolite annotation, for example, *N′,N″,N‴*-tris(dihydrocaffeoyl)spermine, were based on the accurate mass and unique mass fragmentation patterns and is visually demonstrated in Figure 7. The polyamine–cinnamic acid conjugate has previously been reported within plant species of the Solanaceae family in response to wounding **(** Dastmalchi et al., 2014) and viral infection (Rossouw et al., 2019). The mass fragmentation pattern of each metabolite was investigated in both ionization modes, in conjunction with the use of MS^E^ energy ramping within the ESI collision cell. These strategies were used to demonstrate how the MassFragment plugin of the MassLynx XS software eased metabolite annotation and bolstered the verification of well-described compounds. The elemental compositions of fragment ions were also calculated as a secondary method of validating each compound’s structural identity (Figure 7). Finally, the annotation of each metabolite was cross-referenced with the scientific literature.

Many of the phytochemicals identified have been previously reported in publications, dedicated tomato databases, or in related species within the Solanaceae family (Gómez-Romero et al., 2010; Narváez-Cuenca et al., 2013; Roldan et al., 2014; Cichon et al., 2017; Zeiss et al., 2019). The metabolites were tabulated based on the ascending Rt with the corresponding *m/z* values. Several of the identified phytochemicals were members of the phenylpropanoid class, e.g., hydroxycinnamic acids (HCAs), benzoates and flavonoids, conjugated amides of HCAs (HCAAs), conjugated esters of HCAs (chlorogenic acids, CGAs), amino- and organic acid derivatives, and several members of diverse lipid classes.

### 3.8 Semi-quantitative analysis of discriminant oxylipins and benzoyl ornithine

**FIGURE 8:**
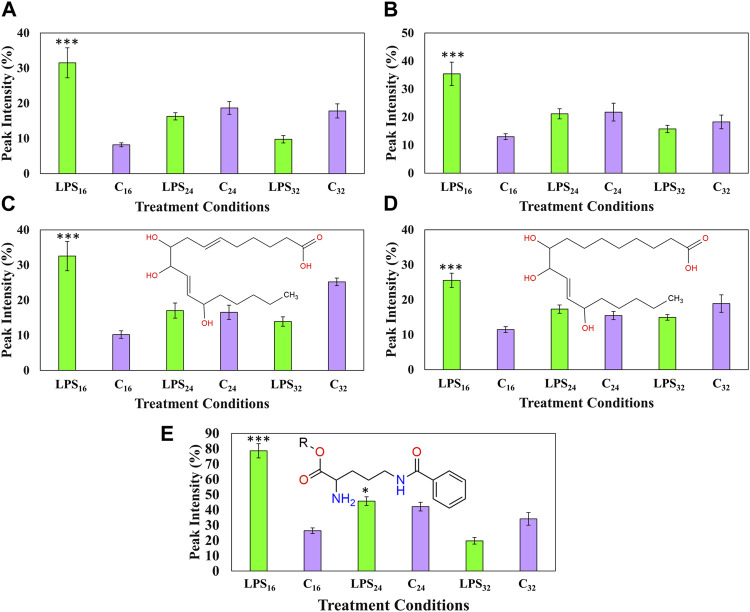
Fluctuating cellular levels of the selected discriminant ions in extracts from tomato leaves in the early response after treatment with LPS_*R. sol.*_ The relative peak intensities of two trihydroxy octadecadienoic acid isomers **(A**, **C)**, two trihydroxy octadecenoic acid isomers **(B**, **D),** and benzoyl ornithine glycoside **(E)** over the described incubation time points (16, 24, and 32 h) are presented in green. MgSO_4_ control (C—highlighted in purple) was included for each time point as a comparative measure. Each data bar is presented as a mean value (*n* = 9 samples) with the error bars indicating the calculated standard deviation (σ). A two-condition paired Student’s t-test was performed to compare the treatments with MgSO_4_ control where the asterisks indicate levels of statistical significance (* = *p* value ≤0.01, ** = *p* value ≤0.001, and *** = *p* value ≤0.0001). R indicates the position of an ester-bonded glycoside group in the benzoyl ornithine derivative (**E**).

Following the metabolite investigation step, the annotated metabolites that exhibited a positive correlation over the described incubation intervals were selected for further downstream analysis. The frequent identification of oxylipins as biomarkers associated with the early host responses to MAMPs and live pathogen infection was recently reviewed (Pretorius et al., 2021). Here, the relative peak intensities of several oxylipin markers were compared between the described groups over the incubation time intervals (Figure 8). An analysis of the fluctuations during the early 16-h response revealed the increased production of four oxylipins, namely, two isomers of trihydroxy octadecadienoic acid (Figures 8A, C) and trihydroxy octadecenoic acid, respectively (Figures 8B, D). Each of the oxylipin molecules demonstrated an increase 16 h aftwe LPS_*R. sol.*_-infiltration followed by a decrease returning to levels comparable to the corresponding control. A two-condition pairwise Student’s t-test was applied, in addition to the previous application of univariate descriptive statistics, to determine the presence of a statistical difference between the groups. In addition, the relative cellular content of a benzoyl ornithine derivative was similarly investigated (Figure 8E). Ornithine is a non-proteogenic amino acid and precursor of putrescine, a polyamine (Zeiss et al., 2021b). The relative abundance of the ornithine compound was found to transiently increase during the 16-h incubation interval followed by a decrease to new cellular homeostatic levels during the 24 and 32 h incubation times (Figure 8E). The synthesis of the abovementioned molecules increased (>two-fold) during the 16-h incubation point, suggestive of either a dedicated role during the early responses of plant signaling and defense or as unanticipated byproducts synthesized throughout the early stages of cellular recognition. Interestingly, the investigated oxylipin molecules, along with several other lipid species, have previously been reported as discriminant markers associated with LPS-treatment (Finnegan et al., 2016; Mareya et al., 2020; Tinte et al., 2020). From a proteomic perspective, several plasma-membrane associated proteins linked to oxylipin synthesis, e.g., phospholipase D, have also been discovered as markers induced by LPS chemotype treatments (Hussan et al., 2020).

### 3.9 Semi-quantitative analysis of discriminant nitrogen-containing metabolites

**FIGURE 9:**
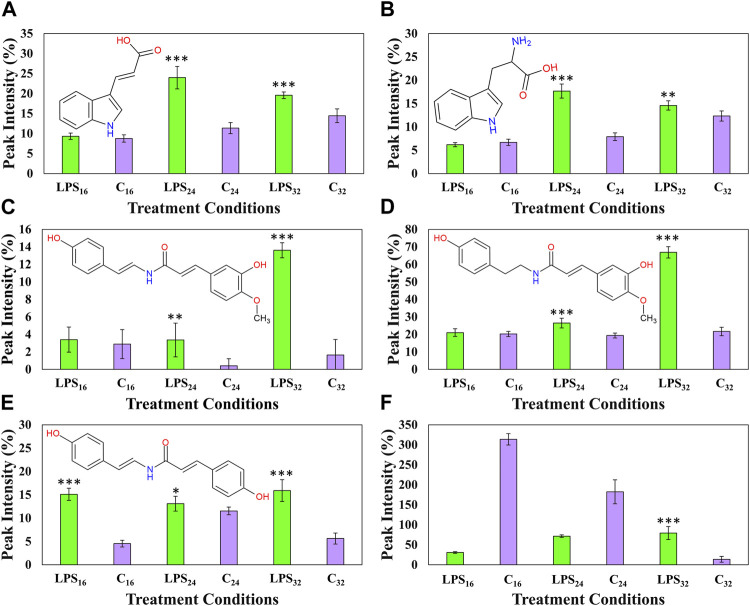
Fluctuating cellular levels of the selected response markers in extracts from tomato leaves after treatment with LPS_*R. sol.*_ The relative peak intensities of indole acrylic acid **(A)**, tryptophan **(B)**, feruloyl dehydrotyramine **(C)**, feruloyl tyramine **(D)**, coumaroyl tyramine **(E)**, and *N′,N″,N‴*-tris(dihydrocaffeoyl)spermine **(F)** over the described incubation time points (16, 24, and 32 h) are presented in green. MgSO_4_ control (C, highlighted in purple) was included for each time point as a comparative measure. Each data bar is presented as a mean value (*n* = 9 samples) with the error bars indicating the calculated standard deviation (σ). A two-condition paired Student’s t-test was performed to compare the treatments with the MgSO_4_ control where the asterisks indicate levels of statistical significance (* = *p* value ≤0.01, ** = *p* value ≤0.001, and *** = *p* value ≤0.0001).

Six nitrogen-containing metabolite features, namely, indole acrylic acid, tryptophan, feruloyl tyramine, feruloyl dehydrotyramine, coumaroyl tyramine, and *N′,N″,N‴*-tris(dihydrocaffeoyl)spermine, were also identified as discriminant ions positively correlated to the LPS_*R. sol.*_ throughout the incubation intervals (Figure 9). The two indole-containing compounds, tryptophan, and indole acrylic acid (Figures 9A,B) were upregulated during the 24- and 32-h incubation intervals. A similar trend was also observed with feruloyl dehydrotyramine (Figure 9C) and feruloyl tyramine (Figure 9D), where the synthesis of both compounds was upregulated during the 24-h interval followed by a large increase in production leading up to the 32-h incubation point. The cellular levels of coumaroyl tyramine were found to increase throughout each of the incubation intervals (Figure 9E). Finally, the synthesis of the conjugated spermine derivative was downregulated during the early and middle incubation points. This might be indicative of reprioritization/reallocation of metabolic reserves or utilization of the HCA and polyamine comprising the conjugate (Zeiss et al., 2021b). Interestingly, it was only found to increase again at the 32-h incubation point (Figure 9F). This trend hints to the compound playing a functional role in the overall recovery of the host’s metabolism following the initial perturbation.

## 4 Discussion

### 4.1 LPS as a microbe/pathogen-derived molecular pattern molecule

Plant hosts are generally exposed to a variety of pathogen-derived MAMPs that can be perceived by the innate immune system (Sanabria et al., 2010; Zhou and Zhang, 2020). The resultant defense response is thus the result of a number of perception and signal transduction events (Boller and Felix, 2009; Malik et al., 2020). By following a reductionist approach with discrete elicitors (Zeiss et al., 2021a; 2022), the contribution of individual MAMPs to the combined response might be elucidated.

LPS perform several roles in the interaction between the bacterial pathogen and its eukaryotic host. In the context of pathogenesis, LPS coating contributes to the exclusion of plant-derived antimicrobial phytoanticipin and phytoalexin molecules (Ranf, 2017). Although the literature has documented the roles of LPS in promoting bacterial survival, the mechanisms surrounding LPS perception during plant–pathogen interactions remain limited (Sanabriavan Heerden and Dubery, 2012; Mareya et al., 2020).

*R. solanacearum* multiplies and spreads through the plant xylem vessels. Due to the surface location of LPSs, high concentrations of the MAMP would reveal the presence to the plant immune surveillance system. However, to date, no dedicated pattern recognition receptor (PRR) responsible for LPS perception in plants has been documented. Assuming analogy to other defense responses, the perception of LPS via membrane-mediated PRRs would be the plausible outcome to generate a biochemical signal via a PRR-RBOHD (respiratory burst oxidase homolog D) module. It is known that LPS can trigger oxidative bursts that are low in intensity but sustained over a long time interval. The literature on *Arabidopsis* has documented the capacity of LPS to trigger two successive ROS bursts (Shang-Guan et al., 2018). Little is known about the mechanism by which LPS is perceived in plants and how this dynamic results in the activation of different plant responses (Ranf, 2017; Tinte et al., 2020). Nonetheless, ROS signaling acts as a central driving force in plant cells by integrating many different signal transduction pathways that would trigger induced phytochemical responses, leading to a final biochemical phenotype in support of plant defense and survival. Interestingly, although LPS represents a major membrane component of potential Gram-negative pathogens, the changes to the metabolome observed in this study are relatively minor or attenuated when compared to that triggered by flagellin-derived peptides flg22 and flg28 under the same experimental conditions (Zeiss et al., 2022).

It is believed that lipid A of LPS from different species lies at the center of the biological activity of LPS (Madala et al., 2011). Certain bacteria can manipulate the composition of their lipid A in response to environmental cues and thereby evade, modulate, or even antagonize the triggering of innate host responses (Munford and Varley, 2006; Madala et al., 2012). Variations in lipid A structures are exhibited by different acylation patterns and the number and location of phosphates, leading to heterogeneity observable even in the same bacterium (Schromm et al., 1998; Holst and Molinaro, 2009). The chemical composition, structure, and conformation of lipid A may thus be important determinants during a plant’s interaction with pathogenic or beneficial bacteria.

Here, we focused on the LPS from *R. solanacearum* that was found to be composed of a mixture of two OPS repeating units, i.e., either a linear tetrasaccharide (as the major component) or a branched xylose-containing pentasaccharide (as the minor component), which was consistent with the previous data (Kocharova et al., 1993; Varbanets et al., 2003). Strikingly, lipid A was found to be hypoacylated, that is, it mainly carries five or four acyl chains, with only minor hexa-acylated forms identified. Of note, and in accordance with previous data reported for *Ralstonia* genus (Varbanets et al., 2003; Zhang-Sun et al., 2019), a high degree of hydroxylation of secondary fatty acids has been observed with 14:0(2-OH) or 16:0(2-OH) found on the reducing glucosamine unit.

This raises the question of whether the low level of lipid A acylation of the *R. solanacearum* pathogens might play a role in i) evasion of immune recognition or ii) dampening the intensity of the triggered defense response. Although, in comparison with human pathogens, much less is known about the strategies used by phytopathogens to escape the plant immune detection, several studies have shown that significant alterations in the lipid A structure resulted in a decreased immunoactivity of the LPS (Di Lorenzo et al., 2022). These structural alterations include the reduction of the acylation degree and the non-stoichiometric substitution of the phosphates with positively charged residues (such as 2-aminoethyl phosphate or Ara4N) (Silipo et al., 2008; Di Lorenzo et al., 2022). In this frame, the underacylation of lipid A has been associated with the abatement of plant defense response, while the occurrence of positively charged groups masking the phosphates are considered a counter measure adopted by phytopathogens to evade plant immune vigilance. As a matter of fact, lipid A from the *R. solanacearum* strain analyzed in this study was hypoacylated and, in minor species, it was also found to be decorated by Ara4N. Therefore, it is tempting to hypothesize that the peculiar lipid A chemical structure of this strain might have a key role in facilitating/enabling bacterial survival and resilience within the host plant. In addition, the expression of two diverse OPS, as previously observed for other *Ralstonia* strains, and both containing a high number of deoxy sugar residues, which render the OPS more hydrophobic, might be considered additional strategies to modulate or suppress plant defense responses, thereby facilitating the establishment of the infection.

Metabolomics tools and approaches have developed to a core technology in the plant sciences that produces large, information-rich datasets. Metabolic profiling using an untargeted UHPLC-MS approach provided adequate levels of resolution and sensitivity required to capture the fine fluctuations frequently observed in the metabolome. In general, plants mobilize similar chemical defense responses as reflected by the activation of similar pathways leading to secondary metabolite synthesis (Zeiss et al., 2021b). At the metabolite level, this might be reflected in enhanced synthesis of secondary metabolites with antimicrobial and antioxidant activities. Moreover, plants execute the triggered defenses based on the perceived stimulus and the existing biochemical background operative in the naïve vs. stress-related conditions. Allowing for the dynamic nature of plant metabolism, qualitative and quantitative differences of specific metabolites or classes of metabolites within the broader metabolomic profiles may modulate the eventual outcome of a host response to attempted infection (Mhlongo et al., 2021). These molecules (Table 1 and discussed as follows) were found to accumulate in varying amounts in the LPS_*R. sol.*_-infiltrated leaves and exhibit varying accumulation patterns. These patterns indicate differential reprogramming over time (either high or low accumulation at specific time points, reflecting early, late, or oscillatory responses). The time-dependent reprogramming is an indication that plants re-adjust their metabolomes toward defense responses in order to ward off infection (Mhlongo et al., 2021). In the absence of a real infection by the *R. solanacearum* pathogen, the levels of the LPS_*R. sol.*_ -induced metabolites decrease with the establishment of a new cellular homeostasis.

### 4.2 Changes in lipidome components

The annotated metabolomics data suggest a positive correlation between the early stages of LPS_*R. sol.*_ treatment, i.e., the 16-h inoculation point and the increased metabolic activity within the oxylipin biosynthetic pathway (as shown in Figure 8). A previous study investigating the changes observed in the metabolome of *Sorghum bicolor* after treatment with LPS from *Burkholderia andropogonis* reported the increased production of triHODE among other identified lipid molecules (Mareya et al., 2020). Our recent review has described the direct involvement of fatty acids and oxylipins in plant defense (Pretorius et al., 2021). Fatty acid peroxidation can occur via enzymatic or non-enzymatic means. However, the intricate process of lipid peroxidation along with the discriminant increase in oxylipin content during the 16-h interval would suggest direct enzymatic action following initial LPS_*R. sol.*_ perception and the subsequent launch of an early immune response from the lipoxygenase pathway. The overall antimicrobial activity of the oxylipins is dependent on various structural and chemical properties, e.g., length of the carbon chain, as well as the presence, number, position, and orientation of double bonds (Deboever et al., 2020). Membrane lipids are often regarded as substrates to produce several signaling molecules such as phosphatidic acid and phosphoinositide species, as well as free fatty acids and phytohormones, where the production of these molecules is typically initiated as a response to stress. Lipids also have auxiliary functions in plant defense, which include acting as structural defense contributors (cell membrane and -wall) and serving as specialized antimicrobial compounds (Mareya et al., 2020). The external signals and underlying mechanisms that mediate the subsequent release and synthesis of these hydrophobic molecules remain an unexplored scientific field. The development of emerging lipidomics in conjunction with the advances in MS instrumentation will have a direct impact on the functional analysis of these lipid molecules in coming years.

### 4.3 Hydroxycinnamic acid amide (HCAA) production

This study demonstrated that the infiltration of LPS_*R. sol.*_ into the leaf tissues resulted in the elevated production of the phenolic conjugates coumaroyl tyramine, feruloyl tyramine, and feruloyl dehydrotyramine during the later stages of the induced response. The induction of these compounds by *R. solanacearum-*derived peptide elicitors (flg22, flgII-28, and csp22) have been previously reported in the same experimental model (Zeiss et al., 2021a; Zeiss et al., 2022). Relatedly, a previous study showed the chitosan-induced production of the abovementioned compounds in tomato, and through fluorescence detection and mutant analysis, it demonstrated that the production of feruloyl tyramine was responsive to the cellular levels of systemin and jasmonic acid (Pearce et al., 1998). The production of coumaroyl tyramine, feruloyl tyramine, and associated conjugates was also observed in the metabolic profiles of *Nicotiana tabacum* cells after treatment with LPS, chitosan, and flg22 (Mhlongo et al., 2016). The presence of the HCAAs in response to biotic stress implicated these secondary metabolites with associated roles in the Solanaceae host defense system. The HCAAs are nitrogen-containing molecules synthesized by the enzymatic action of hydroxycinnamoyl transferase(s) that act on the free amine groups of either polyamines or aromatic amines, and the activated thioester (Co-A) derivatives of the HCAs, the latter precursors are synthesized *via* the early phenylpropanoid pathway (Zeiss et al., 2021b). Moreover, the detection and elevated production of the benzoyl ornithine derivative points to the activation of the polyamine biosynthetic pathway. HCAAs are regarded as the end products of polyamine and aromatic amine metabolism that create a metabolic storage pool to modulate the flux of both parental constituents. The conjugation of the two precursors alters the overall characteristics of the product molecules. These altered chemical properties facilitate the translocation, chemical stability, and compartmentalization of the HCAAs within the host cells (Zeiss et al., 2021b).

In addition to their antimicrobial activity as soluble phenols, HCAAs have also been proposed to crosslink structural polymers in the cell wall during infection, potentially contributing toward the formation of a phenolic barrier that can make the cell wall more resilient to pathogenic degradation (Zeiss et al., 2021b). Relatedly, cell wall defenses have been identified as important as inducible barriers against infection and spreading of *R. solanacearum* (Shi et al., 2023). A recent paper reported that tomato varieties tolerant to virulent strains of *R. solanacearum* have the ability to restrict bacterial movement and to slow disease progression, thus enhancing apparent resistance to the pathogen (Kashyap et al., 2022). These resistant tomato cvs. specifically responds to infection by assembling a vascular structural barrier formed by a ligno-suberin coating and tyramine-derived HCAAs.

## 5 Conclusion

Detection of infection by microbial phytopathogens through immune receptors triggers signaling cascades that initiates dynamic and interconnected, multi-level defense responses. To date, little is known about the underlying mechanisms that mediate LPS perception in tomato and other plants. We have previously argued that the lipid and glycan molecular patterns of the LPS molecule act as partial agonists, but that the intact LPS structure is required for the full agonist activity. The structural analysis of the LPS_*R. sol.*_ points to the composition and acylation pattern of lipid A as a mechanism by which the pathogen may attempt to evade immune recognition or to dampen the intensity of the MTI defense response, as well as alter the dynamics that result in the activation of downstream plant defense strategies. Overall, the results generated from this untargeted metabolomics approach demonstrated that LPS_*R. sol.*_ perception in *S. lycopersicum* leads to a redirection of cellular metabolism in support of producing several defense-related metabolites such as the HCAs, HCAAs, and oxylipins. By comparison, few flavonoids and steroidal glycoalkaloids were identified as discriminatory biomarkers. Specific metabolites identified as potential biomarkers for future studies were from the HCAA class which included feruloyl tyramine, coumaroyl tyramine, as well as indole acrylic acid and the aromatic amino acid, tryptophan as the precursor molecule of secondary metabolites. Various studies have documented the production of HCAAs in tomato and other species of the Solanaceae family in response to biotic stress or elicitor treatments, suggesting that these molecules have dedicated functional roles during plant–pathogen interactions. The HCAAs clearly play a significant role in the defense response of tomato and may be determining factors in the resistance response to *R. solanacearum* infection. Several oxygenated fatty acid derivative molecules, namely, trihydroxy octadecadienoic acid and trihydroxy octadecenoic acid, were also found to be upregulated in tomato during the early stages of LPS_*R. sol.*_ treatment. The oxylipins were also identified as potential markers within the lipid class that can be studied in future research relating to plant defense within the Solanaceae family. The increased production of the lipid molecules during elicitor treatment is suggestive of the active involvement of oxylipins during host perception, signal transduction, and plant defense. Some of the abovementioned compounds have been reported in the scientific literature, but the inducers and cellular dynamics by which these molecules contribute to the overall plant defense in the tomato, *R. solanacearum* pathosystem, remain largely unknown.

## Data Availability

The datasets presented in this study can be found in online repositories. The study design information, LC-MS raw data, analyses, data processing information, and the meta-data were deposited to the EMBL-EBI metabolomics repository MetaboLights as MTBLS7324, https://www.ebi.ac.uk/metabolights/MTBLS7324.
