# Supplementary material for: Lipopolysaccharides from Ralstonia solanacearum induce a broad metabolomic response in Solanum lycopersicum
Source: Front Mol Biosci. 2023 Aug 10;10:1232233. doi: 10.3389/fmolb.2023.1232233 (PMC10450222; doi:10.3389/fmolb.2023.1232233)
Supplement: Supplementary file 1 [file Table1.DOCX]

**Lipopolysaccharides from *Ralstonia solanacearum* Induce a Broad Metabolomic Response in *Solanum lycopersicum***

Dylan, R. Zeiss^1^, Antonio Molinaro ^2,3^, Paul A. Steenkamp^1^, Alba Silipo^2,3^, Lizelle A. Piater^1^, Flaviana Di Lorenzo ^2,3^ and Ian A. Dubery^1^*

^1^ Research Centre for Plant Metabolomics, Department of Biochemistry, University of Johannesburg, P.O. Box 524, Auckland Park 2006, South Africa;

^2^ Department of Chemical Sciences and ^3^ Task force on Microbiome studies, University of Napoli Federico II, Complesso Universitario Monte Sant’Angelo, Via Cinthia 4, 80126, Napoli, Italy.

**Supplementary data – (Figures S1 – S4 and Tables S1 and S2)**

**Table S1.** ^1^H, and ^13^C (*Italic*) chemical shifts of the OPS derived from mild acid hydrolysis of the LPS from *R. solanacearum*. The chemical shifts of the NH of the acetyl group (*N*Ac) were at ^1^H/^13^C 1.96/22.3 ppm.

|  |  | **1** | **2** | **3** | **4** | **5** | **6** | **7** |
| --- | --- | --- | --- | --- | --- | --- | --- | --- |
| **A** | ^1^H | 5.07 | 3.99 | 3.85 | 3.40 | 3.66 | 1.25 |  |
| 2-α-L-Rha | ^13^C | *100.9* | ***77.8*** | *70.1* | *71.2* | *69.3* | *16.7* |  |
| **B’** | ^1^H | 4.91 | 4.18 | 3.78 | 3.63 | 3.63 | 1.18 |  |
| 3,4-α-L-Rha | ^13^C | *101.7* | *69.8* | ***74.6*** | ***79.4*** | *69.3* | *16.7* |  |
| **B** | ^1^H | 4.89 | 4.22 | 3.77 | 3.43 | 3.63 | 1.18 |  |
| 3-α-L-Rha | ^13^C | *101.7* | *69.7* | ***79.8*** | *71.8* | *69.3* | *16.7* |  |
| **C** | ^1^H | 4.77 | 3.75 | 3.72 | 3.46 | 3.95 | 1.16 |  |
| 3-α-L-Rha | ^13^C | *101.1* | *70.7* | ***77.4*** | *71.5* | *69.1* | *16.7* |  |
| **D** | ^1^H | 4.66 | 3.80 | 3.53 | 3.48 | 3.40 | 3.70/3.85 |  |
| 3-β-D-GlcNAc | ^13^C | *102.0* | *55.8* | ***81.6*** | *68.3* | *75.8* | *60.6* |  |
| **E** | ^1^H | 4.34 | 3.41 | 3.33 | 3.50 | 3.90/3.19 |  |  |
| t-β-L-Xyl | ^13^C | *103.5* | *73.2* | *76.1* | *69.3* | *65.0* |  |  |
| **X** | ^1^H | 5.28 | 3.95 | 3.93 | 3.61 | n.d. | 4.02 | 3.71 |
| 2,3-α-L,D-Hep | ^13^C | *100.0* | ***80.0*** | ***72.4*** | *72.0* | *n.d.* | *68.4* | *63.4* |
| **Y** | ^1^H | 4.75 | 3.96 | n.d. | 3.41 | 3.67 | 1.17 |  |
| *t*-α-L-Rha | ^13^C | *102.6* | *70.4* | *n.d.* | *71.1* | *69.3* | *16.7* |  |

**Figure S1.** Negative-ion MALDI MS/MS spectrum of precursor ions at *m/z* 1505.6, chosen as a representative ion peak of the cluster ascribed to *mono*-phosphorylated penta-acylated lipid A species from *R. solanacearum* LPS. The assignment of main fragments is reported in the spectrum. The proposed structure for the lipid A species is sketched in the inset.


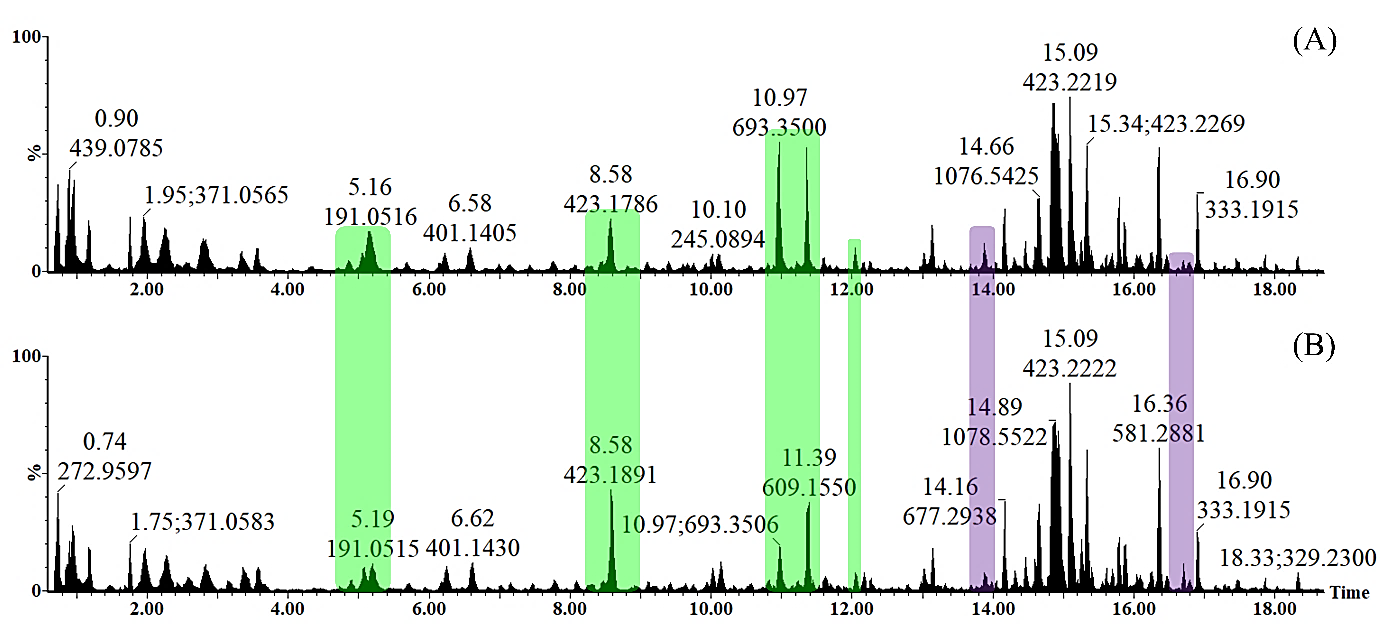


**Figure S2.** Base peak intensity chromatograms of the ESI negative UHPLC-MS analyses of the methanol extracts from the LPS*_R.sol_* (100 µg/mL) inoculated *S. lycopersicum* leaf tissues. A comparison of the metabolite profiles at the 24 h time interval – **(A)** MgSO_4_ negative control and **(B)** LPS treatment - revealed concentration-linked variation in relative peak intensities. The *y*-axes of the two chromatograms are linked and represent the relative abundance (%) of the metabolite signatures at their respective retention times (Rt, min). The changes in peak intensities (green) and/or the presence/absence of peaks (purple) could be observed, reflecting the LPS*_R.sol_* -induced perturbation of leaf metabolism.


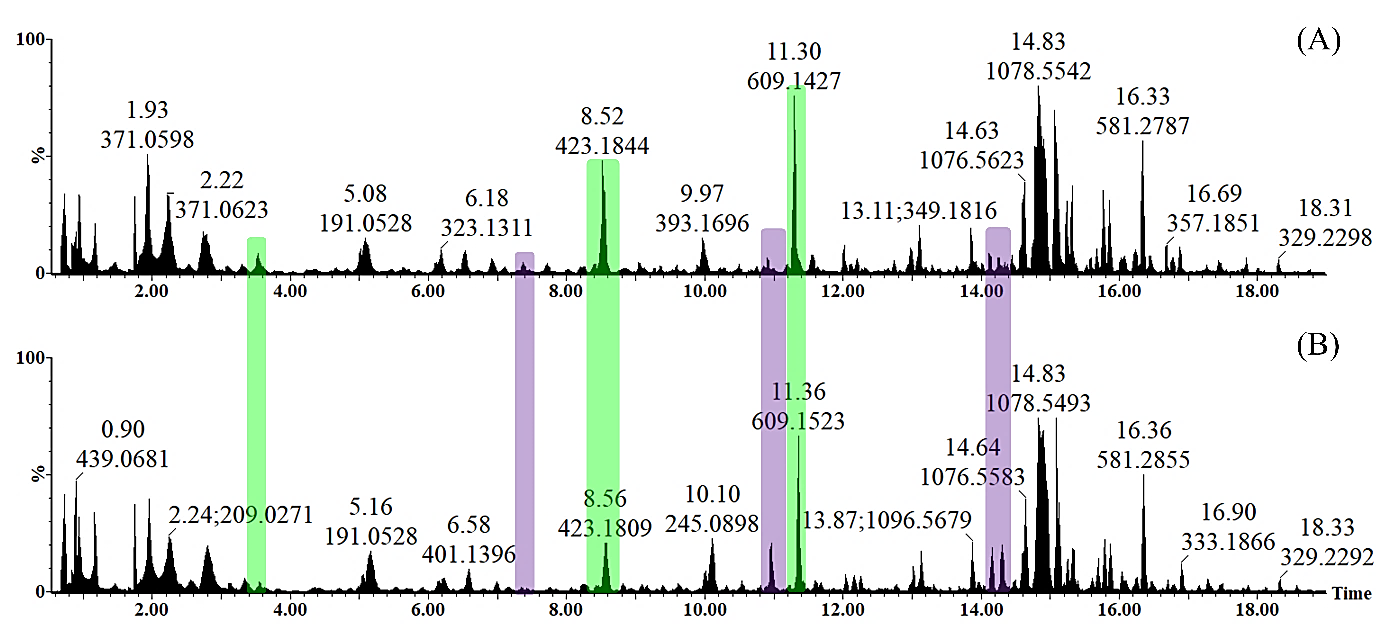


**Figure S3.** Base peak intensity chromatograms of the ESI negative UHPLC-MS analyses of the methanol extracts from the LPS*_R.sol_* (100 µg/mL) inoculated *S. lycopersicum* leaf tissues. A comparison of the metabolite profiles at the 32 h time interval – **(A)** MgSO_4_ negative control and **(B)** LPS treatment - revealed concentration-linked variation in relative peak intensities. The *y*-axes of the two chromatograms are linked and represent the relative abundance (%) of the metabolite signatures at their respective retention times (Rt, min). The changes in peak intensities (green) and/or the presence/absence of peaks (purple) could be observed, reflecting the LPS*_R.sol_* -induced perturbation of leaf metabolism.


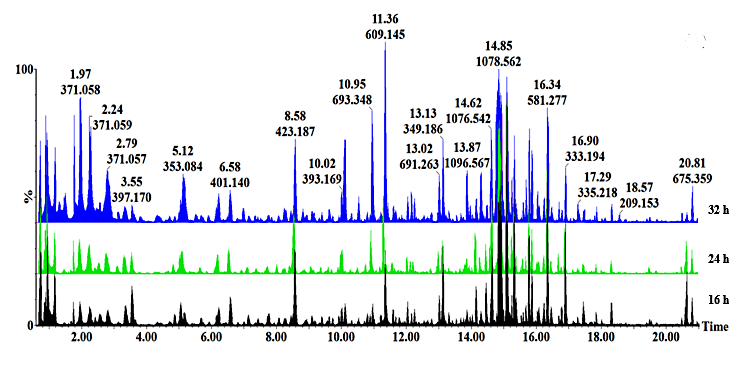


**Figure S4.** Overlaid UHPLC-MS BPI chromatograms (ESI^-^) of methanolic leaf extracts from the LPS*_R. sol_* elicitor treated tomato plants after 16 h (black), 24 h (green) and 32 h (blue). The chromatograms highlight time-dependent metabolic variations as a result of LPS treatment. Qualitative differences are reflected by the peak intensities where the *y*-axis represents the relative peak intensity of the metabolites at their respective retention times.

**Table S2.** Statistical validation of the computed OPLS-DA models corresponding to the LPS elicited tomato leaf treatment data matrices. The calculated number of components used in each final model (N), the R^2^X(cum), the R^2^Y(cum) and the Q^2^(cum) values for each of the six OPLS-DA models are presented for both ESI negative and ESI positive modes. The R^2^ and Q^2^ values of the permutation analysis (*n =* 200 random permutations) are compared and shown to be significantly lower than the original values. Model values > 0.50 are shaded in green, values < 0.50 but > 0.30 are shaded in orange, and values < 30 are shaded in red. The *p-*value of a 7-fold CV-ANOVA was shown to indicate statistical significance of each investigated model. (AUROC = Area Under the Receiver Operating Characteristics curve; LPS*_R. sol_* = *R. solanacearum*-derived lipopolysaccharide treatment; all at time intervals of 16 h, 24 h and 32 h post-elicitation; 8 mM MgSO4 controls at the same time points).

| **Model** | **N** | **R^2^X**  **(cum)** | **R^2^Y**  **(cum)** | **Q^2^**  **(cum)** | **Permutation** | | **AUROC** | | ***p*-value of CV-ANOVA** |
| --- | --- | --- | --- | --- | --- | --- | --- | --- | --- |
|  |  |  |  |  | **R^2^** | **Q^2^** | **Control** | **LPS*_R. sol_*** |  |
| ESI (Negative) Supervised Models | | | | | | | | | |
| C__16_ *vs.* LPS_16_ | 17 | 0.406 | 0.998 | 0.970 | 0.828 | -0.299 | 0.906 | 1.000 | 5.337 x 10^-9^ |
| C__24_ *vs*. LPS_24_ | 18 | 0.456 | 0.999 | 0.916 | 0.963 | -0.382 | 0.542 | 0.946 | 2.605 x 10^-5^ |
| C__32_ *vs*. LPS_32_ | 16 | 0.363 | 0.997 | 0.945 | 0.904 | -0.320 | 1.000 | 0.690 | 7.348 x 10^-7^ |
| ESI (Positive) Supervised Models | | | | | | | | | |
| C__16_ *vs.* LPS_16_ | 18 | 0.582 | 0.999 | 0.989 | 0.806 | -0.344 | 0.882 | 1.000 | 1.033 x 10^-12^ |
| C__24_ *vs*. LPS_24_ | 18 | 0.558 | 0.992 | 0.963 | 0.796 | -0.389 | 0.874 | 0.816 | 3.654 x 10^-9^ |
| C__32_ *vs*. LPS_32_ | 18 | 0.467 | 0.995 | 0.697 | 0.797 | -0.385 | 0.689 | 0.545 | 1.556 x 10^-9^ |
